# Moderating effects of self-defined sexual orientation on the relation between social factors and depressive symptoms or suicidal ideation among French young adults

**DOI:** 10.1007/s00127-025-02951-y

**Published:** 2025-06-23

**Authors:** Junko Kose, Camille Davisse-Paturet, Anne Pastorello, Laurence Meyer, Maria Melchior, Cécile Vuillermoz, Alexandra Rouquette, the EpiCov Study Group, Josiane Warszawski, Nathalie Bajos, Guillaume Bagein, François Beck, Emilie Counil, Florence Jusot, Nathalie Lydié, Claude Martin, Philippe Raynaud, Ariane Pailhé, Delphine Rahib, Patrick Sillard, Alexis Spire

**Affiliations:** 1 Université Paris-Saclay, Inserm, Université Versailles Saint-Quentin (UVSQ), Centre d’Epidémiologie et de Santé Publique (CESP), Paris, France; 2 Social Epidemiology Research Team, Institut Pierre Louis d’Epidémiologie Et de Santé Publique (IPLESP), Université, InsermParis, Sorbonne France; 3 Epidemiology and Public Health Department, Assistance Publique-Hôpitaux de Paris Université Paris-Saclay, Le Kremlin-Bicêtre, France

**Keywords:** Depressive symptoms, Epidemiology, Sexual orientation, Social factors, Suicidal ideation, Young adults

## Abstract

**Purpose:**

Disparities in mental health across sexual orientation groups and among young adults have long been discussed. The aim of this cross-sectional study was to investigate the moderating effects of sexual orientation on the associations between social factors and depressive symptoms as well as suicidal ideation in young adults.

**Methods:**

The study included 6,337 participants aged 18–25y in 2022 from the French EpiCov cohort. The outcome variables were depressive symptoms and suicidal ideation. Poisson regressions with robust error variance were performed to investigate the associations between social factors and outcomes according to sexual orientation (lesbian, gay, bisexual, other, or not defining themselves according to their sexuality: sexual minority (SM); heterosexual or not wishing to answer: Not belonging to SM (NSM)).

**Results:**

The prevalence of depressive symptoms and suicidal ideation was higher in the SM than in the NSM group. Regarding depressive symptoms, significant moderating effects of sexual orientation were observed for female vs male sex (NSM: adjusted Prevalence Ratio (aPR) 1.58[1.28–1.95], SM: aPR 1.03[0.78–1.36]) and age category 22–25y vs 18-21y (NSM: aPR 1.32[1.05–1.67], SM: aPR 0.78[0.59–1.03]). Regarding suicidal ideation, significant moderating effect was observed for not being vs being in a relationship (NSM: aPR 1.55[1.14–2.12], SM: aPR 0.82[0.59–1.13]).

**Conclusion:**

In this study conducted in 2022, well-known social risk factors of mental problems do not explain the higher prevalence of depressive symptoms and suicidal ideation among young SM group. Further studies are needed to understand the specific challenges faced by these young people.

**Supplementary Information:**

The online version contains supplementary material available at 10.1007/s00127-025-02951-y.

## Introduction

Individuals identifying within the sexual minority community (individuals who identify as part of sexual orientation diverse communities) have long faced higher risks of mental health conditions and suicidal thoughts and behaviors than their heterosexual counterparts [1–3]. In 1995 for gay men and in 2003 for lesbian, gay, and bisexual people, Ilan Meyer described a conceptual framework for understanding this higher risk, called “Minority stress theory” [4, 5]. The framework includes proximal stress (internal vigilance and concern about discrimination, internalized stigmatization about sexuality, and concealment or disclosure of sexual orientation) and distal stress (stress due to interpersonal relationships, such as experiences of rejection or discrimination by others) [4, 5]. In that sense, the stress faced by the sexual minority community should be considered not only as an individual attitude, but also as a consequence of the social and political climate toward this community [6]. Indeed, following a joint statement by the United Nations agencies in 2015 to condemn violence and discrimination against LGBTQI + people (lesbian, gay, bisexual, transgender, queer, intersex, and inclusive of individuals who identify as part of sexual and gender diverse communities), the United Nations Secretariat adopted in 2024 a strategy to protect them from violence and discrimination [7]. While there is ongoing progress to support the rights of members of this community worldwide, same-sex relationships remain a crime in many countries, and decriminalization or moving sexual orientation out of the psychiatric field are relatively recent in other countries. For example, the American Psychiatric Association included the diagnosis of “homosexuality” in the Diagnostic and Statistical Manual until 1974 [8]. Regarding the World Health Organization, although “homosexuality” has been removed from the International Classification of Diseases (ICD) since 1992, the International Classification of Diseases (ICD)-10 still included “Psychological and behavioral disorders associated with sexual development and orientation” suggesting mental disorders uniquely linked to sexual orientation [9]. This category was removed from the ICD-11, which officially entered into force in 2022. In France, discriminatory laws regarding the age of consent for sexual intercourse (15 years for the opposite sex and 18 years for the same sex) had existed, and “homosexuality” could be grounds for termination of a residential lease until 1982 [10]. The civil solidarity pact (Pacte civil de solidarité: PACS) for same-sex couples was approved in 1999, however, it did not permit joint adoptions by same-sex couples [11]. Marriage for same-sex couples, which allows for joint adoption by same-sex married couples, was approved in 2013 (Law n°2013–404). These transitions may lead to generational differences in the associations between sexual orientation and mental health [12]. As regards societal attitude towards the sexual minority community, French interministerial delegation mobilized against hate reported that since 2016, anti-LGBT + crimes and misdemeanors have increased by 129% and 115% of increase for ‘anti-LGBT + ’ contraventions [16]. The majority of recorded anti-LGBT + crimes affected young people under the age of 30 [16]. In addition, mental disorders account for a greater percentage of the overall burden of diseases in young adults in European high-income countries [13]: among them, depressive disorders are the first leading cause of Disability Adjusted Life Years [14]. Likewise, suicide and “intentional self-harm” is the second leading cause of death in young people in Europe [15]. Continuous assessment of these associations in younger generations is therefore important to inform and adapt public health strategies to the challenges actually faced by these new generations.

In general, mental health conditions result from a complex interplay of social, psychological, and biological factors. For instance, female sex, low income, low socioeconomic status, being single, being from ethnical minorities, and stigmatization have been suggested as risk factors for mental health problems [17–19]. Regarding the sexual minority community, studies have also reported that young age, female sex, low educational attainment, not being in a relationship, and not feeling accepted by others could heightened the risk of depressive symptoms and/or suicidality in this community [20–22]. It should be noted that these studies were conducted using convenient study samples from members of the sexual minority community, which can generate selection bias and do not allow us to investigate whether these factors explain their high risk for depression and suicidality compared to those not belonging to this community.

Previous studies have explored the moderating effect of sexual orientation on the associations between various social factors and depressive symptoms and suicidal thoughts. Indeed, belonging to the sexual minority community has been reported to have strong correlations with both social factors such as discrimination and stigmatization in the family and public contexts [23] and mental health problems [1–3]. The results from previous studies on the moderation effects of sexual orientation suggest that the associations between depressive symptoms or suicidal thoughts and low income, living inner cities, and educational attainment were greater among people belonging to the sexual minority community than among their heterosexual counterparts [24–26]. These studies were conducted in a nationally representative sample of US female elderly using data measured in 2015 [25], in a nationally and regionally representative sample of 8,455 UK adults using data measured in 2008–2013 [26], and in a nationally representative sample of 35,230 US adults using data measured in 2004–2005 [24], respectively. On the other hand, other studies have suggested that victimization, bullying, social support, and childhood gender nonconformity have weaker associations with depressive symptoms or suicidality in participant defined as sexual minority community than in their heterosexual counterparts [27–30]. These findings were obtained in a sample of 3,062 Chinese high school students using data measured in 2018 [28], in a sample of 75,344 US adolescents using data measured in 2009–2011 [27], in a national representative sample of 4,121 US elderly using data measured in 2005–2015 [30], and in a sample of 10,655 US adolescents to young adults using data in measured 2007–2010 [29], respectively. Regarding other social factors, representative household surveys of English adults (N = 10,443) in 2007 and 2014 reported no interaction between gender and sexual orientation on the association with suicidality [31]. Likewise, a Canadian study in a nationally representative sample of 169,091 adults using data in 2007–2017 reported no interaction between sexual orientation and rurality on suicidal behaviors [32]. Overall, the moderating effects of sexual orientation on the associations between social factors and mental health are inconclusive and depends on studied factors, population, geographical context, and period. That may be due to the fact that the effect of sexual orientation on mental health depend greatly on the social and political climate toward sexual minority community [6]. Hence, a comprehensive study of this target population using recent data is necessary.

In France, the latest data on mental health according to sexual orientation were collected before the COVID-19 pandemic. A French study of a nationally representative sample aged 18–69 y in 2012–2019 reported that having same-sex partners was associated with an increased risk of depressive symptoms and suicide attempts [33]. Another French national survey conducted in 2017 among adults 18–75 y revealed that lesbian, gay, and bisexual participants had increased odds of depression, suicide thoughts and attempts compared to heterosexual individuals. This study also reported that 25.4% of the associations were explained by physical or verbal victimization [34], indicating that almost 75% of the associations could be due to other factors. Investigating the moderating effect of sexual orientation on the associations between known social risk factors and depressive symptoms or suicidal thoughts could help understand how such factors explain disparities in mental health across sexual orientation groups.

In this context, the aim of the present study was to investigate whether sexual orientation moderate the associations between various social factors and depressive symptoms as well as suicidal ideation, after verifying the associations between sexual orientation and each outcome, using a recent and large sample of young adults from the French general population, to understand the challenges faced by this new generation of emerging adults.

## Materials and methods

### Study population

The EpiCov (French acronym for “*Epidemiologie et conditions de vie sous le COVID-19”*) French cohort was initiated in 2020, randomly selecting 371,000 participants aged ≥ 15 y in 2020, living in mainland France or in three overseas territories (Martinique, Guadeloupe, and Reunion) from the national tax database (96.4% coverage). The sampling frame was designed to overrepresent underprivileged populations to ensure sufficient statistical power to study social inequalities. People living in medical retirement homes or prisons were not invited to participate in the study [35]. Four waves of data collection were conducted and the present cross-sectional analysis is principally based on the fourth wave, which took place between September 12 and December 5, 2022. Self-computer-assisted web interviews (CAWIs) or computer-assisted telephone interviews (CATIs) was randomly assigned to each participant to collect the data. At the fourth wave, a total of 84,492 participants completed the questionnaire; among them, we included participants aged 18–25 y in 2022 in this study.

All participants provided informed consent to participate in the EpiCov study, which received approval from an ethics committee (*Comité de Protection des Personnes Sud Méditerranée* III 2020-A01191-38) and from France’s National Data Protection Agency (*Commission Nationale Informatique et Libertés,* CNIL, MLD/MFI/AR205138).

### Data collection

All information described below was based on data collected during the fourth wave in September–December 2022, where not specified.

### Moderator: self-defined sexual orientation

Self-defined sexual orientation data were collected using the following question: currently, you define yourself as 1) heterosexual, 2) homosexual, gay or lesbian (people who have sexual relations with people of the same sex), 3) bisexual (people who have sexual relations with people of the same or opposite sex), 4) you don't define yourself by your sexuality, 5) other, 6) you do not wish to answer. Importantly, this question was only asked to adult participants who reported not living with a partner at the fourth wave. The authors of the present work decided not to derive the definition of sexual orientation from the sex of the partner for participants living with a partner, to avoid misclassification bias. For example, a person can self-identify as bisexual and be currently living with a same-sex partner. The analyses were therefore restricted to participants who answered the question on self-defined sexual orientation, thus who were living without partners. Participants defining themselves as homosexual, bisexual, other, or not defining themselves by their sexuality were categorized into the Sexual Minority (SM) group in the present study. Thus, participants who defined themselves as heterosexual or not wishing to answer were categorized into the Not belonging to Sexual Minority (NSM; reference group). Participants who reported not defining themselves by their sexuality were grouped with those who reported being homosexual, bisexual, and other as they shared the same distributions of sociodemographic and health characteristics. Likewise, participants reporting not wishing to answer were grouped with participants reporting being heterosexual, given similarity of their sociodemographic and health-related characteristics. Notably, the prevalence of depressive symptoms and suicidal ideation was low in those who did not wish to response to the question on sexual orientation. The distribution of sociodemographic and health characteristics according to detailed sexual orientation is presented in Supplementary Table S1.

### Outcomes: depressive symptoms and suicidal ideation

Depressive symptoms within 15 days before the response date were assessed using the 9 items of the Patient Health Questionnaire (PHQ-9) [36]. Score ranges from 0 to 27 and a score ≥ 10 was considered as having moderate to severe depressive symptoms (yes/no) [37]. This cut-off score was the most used score in PHQ-9 validation studies and optimal for detecting major depressive disorder [37]. Suicidal ideation was assessed using the following question: “In the last 12 months, have you thought about killing yourself by suicide (yes/no)?”.

### Exposures: social factors

On the basis of prior evidence, the following well-known social factors associated with depressive symptoms and suicidality [38, 39] were selected: sex (female vs male, data from the National Institute for Statistics and Economic Studies: INSEE), age category (22–25 vs 18–21 y), educational attainment (≤ high school vs > high school), employment status (not being employed vs being employed), perceived financial difficulties (yes vs no), being in a relationship (no vs yes), and living alone (yes vs no). The urban density of living area in 2018 (rural = reference; intermediate; high or Paris area) was obtained from INSEE. We also included self-reported history of discrimination in the last 5 years (yes vs no). The questions and response modalities are presented in Supplementary Table S2.

### Covariates

Covariates included the presence of self-reported chronic health conditions or disabilities and a history of mental disorder diagnosis, which are not expected to influence some exposures but are associated with both the moderator and outcomes. We also included the questionnaire type (CAWIs; CATIs) of the fourth wave to account for potential desirability bias due to the survey mode. The questions and response modalities are presented in Supplementary Table S2.

### Statistical analyses

**Fig. 1:**
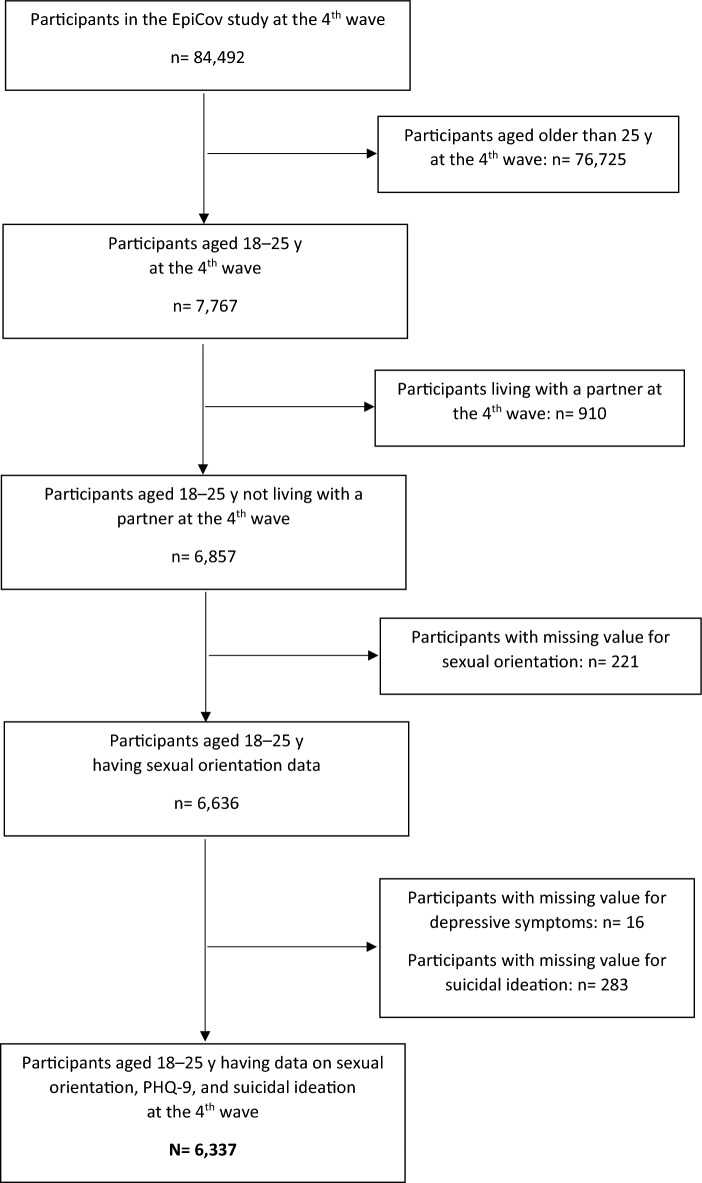
Flowchart of participants

We excluded participants with missing data on outcomes and/or sexual orientation, which are the main variables of interest in the present study. Thus, a total of 6,337 participants aged 18–25 y was included in the analyses (Fig. 1). The comparison of characteristics between the included and excluded participants is presented in Supplementary Table S3. Briefly, the included participants were more likely to be male, 22–25 y, have suicidal ideation, and less likely to have depressive symptoms than those excluded from the analyses. The characteristics of the included participants are presented in the full analyzed sample and according to sexual orientation (Table 1), depressive symptoms (Supplementary Table S4), and suicidal ideation (Supplementary Table S5).

As preliminary analyses, the multiplicative interaction between sexual orientation (moderator) and each social factor (exposure) on the association with each outcome was tested using Poisson regression models with robust error variance [40] to identify interaction terms that would be included in the main model (p < 0.2, described below). The Poisson regression model with robust error variable allows to estimate prevalence ratio, which can address issues of overestimation of associations estimated by Odds Ratios in logistic regression while also being more straightforward to interpret [41]. Each model was run, including all exposures, questionnaire type, and an interaction term between sexual orientation and each exposure. The results for each model are presented using a common exposure reference group (no risk factor and NSM group) to present the adjusted Prevalence Ratio (aPR) of each category according to social factors and sexual orientation, as recommended by VanderWeele et al. [42] (Supplementary Figure S1 and S2). This presentation allows us to examine adjusted associations between sexual orientation and each outcome that cannot be observed in stratified analyses by sexual orientation (i.e., main analyses, see below).

**Fig. 2:**
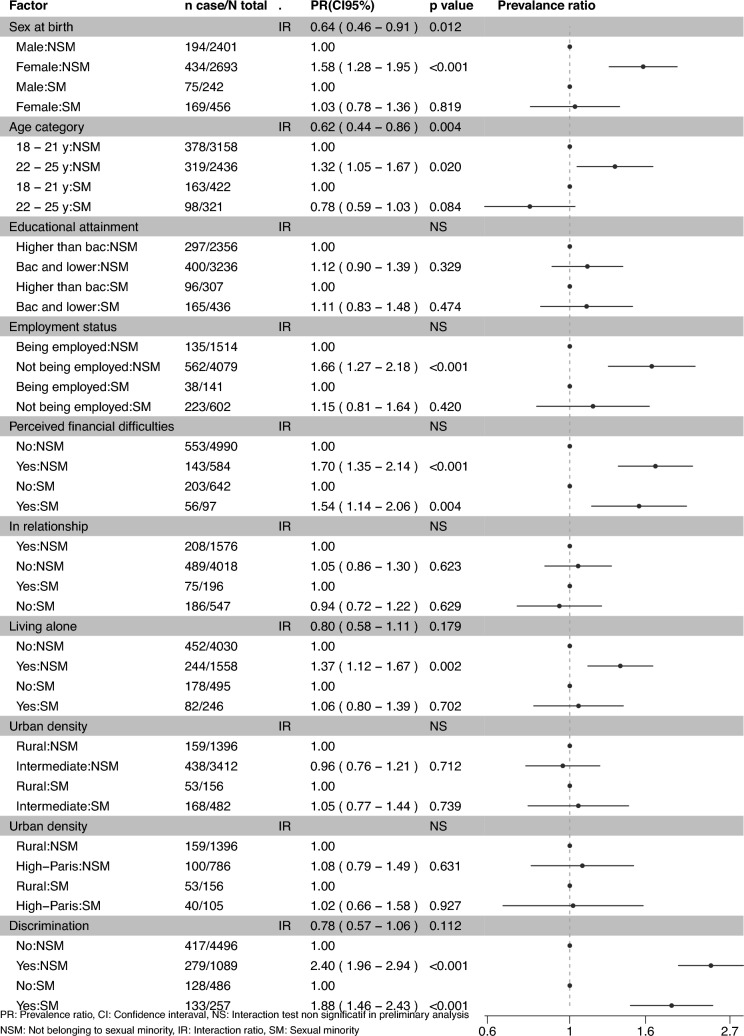
Associations between social factors and depressive symptoms according to sexual orientation (N = 6,337 aged 18–25y; EpiCov study in 2022; weighted and pooled)

**Fig. 3:**
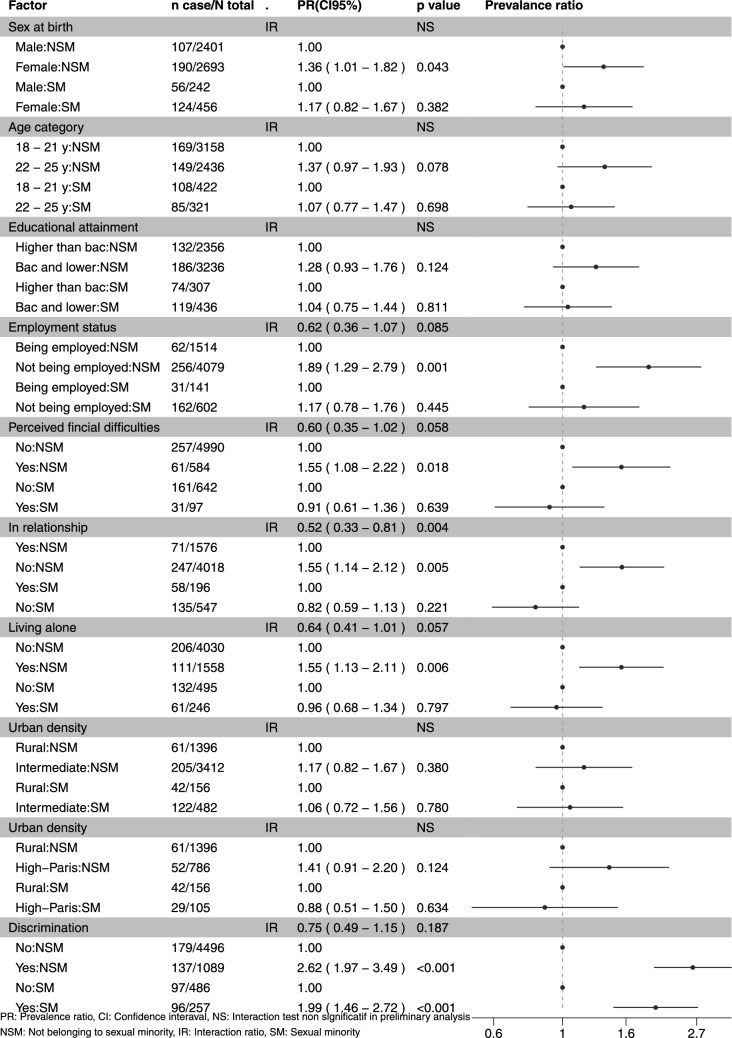
Associations between social factors and suicidal ideation according to sexual orientation (N = 6,337 aged 18–25y; EpiCov study in 2022; weighted and pooled)

For the main analyses, we first run a Poisson regression model with robust error variance for each outcome including all exposures, thus mutually adjusted, questionnaire type, and interaction terms between sexual orientation and exposures having p < 0.2 in the preliminary analyses to obtain adjusted Interaction Ratio (IR). IR is calculated by dividing the observed joint effect of the moderator and exposure by the multiplication of their independent effects, which correspond to the exponentiate of beta coefficient of interaction term in regression models [42]. When the moderator and exposure increase the risk of outcome, an IR > 1 indicates a positive multiplicative interaction (observed joint effect is greater than expected), while an IR < 1 indicates a negative multiplicative interaction (observed joint effect is smaller than expected). When the IR is equal to 1, there is no multiplicative interaction [42]. Afterwards, we ran a Poisson regression model with robust error variance for each outcome, including all exposures and questionnaire type, stratified by sexual orientation to obtain aPR for each exposure according to sexual orientation (Fig. 2 and 3).

In the sensitivity analyses, we repeated the procedure described above by adding chronic health conditions and a history of mental disorder diagnosis as covariates to the main model in order to take into account their potential effect on the associations between social factors and depressive symptoms or suicidal ideation (Supplementary Figure S3–**S6**). An additional sensitivity analysis was conducted excluding participant who did not wish to report their sexual orientation to consider potential differences from participants who reported a heterosexual orientation (Supplementary Figure S7–**S10**). For the preliminary, main, and sensitivity analyses, social factors and/or covariates with missing values were imputed by Multivariate Imputation by Chained Equations assuming that data were missing at random (10 imputation sets: 10.8% in the study sample had missing data on at least one social factor) [43].

Data management was performed using SAS v9.4. Missing value imputation, descriptive, bivariate, and regression analyses were performed using R 4.0. Study weights were applied to descriptive, bivariate, and regression analyses to account for EpiCov’s survey design and non-participation bias (detailed elsewhere [44]). In brief, survey weights were based on demographic and socioeconomic indicators obtained from the FIDELI database and EpiCov previous waves that may be relevant to response probabilities. The margins were also adjusted for general population based on census data and population projections. All tests were two-sided and p < 0.05 was considered as evidence for statistical significance.

## Results

### Characteristics of the participants

Among 6,337 participants of the fourth wave of the EpiCov study, aged 18–25 y with available data on sexual orientation and both study outcomes, 11.0 [10.1–12.1] % belonged to the SM group, and 15.1 [14.0–16.3] % and 7.4 [6.7–8.2] % presented depressive symptoms and suicidal ideation, respectively. 45.6 [43.9–47.3] % were female and 38.4 [36.8–39.9] % had an educational attainment higher than high school. Participants in the SM group were more likely to be women, live alone, experience discrimination, have chronic health conditions, and have been diagnosed with mental disorders than their counterparts. They were more likely to experience depressive symptoms (33.9 [29.6–38.4] %) and suicidal ideation (23.0 [19.5–26.8] %) than those in the NSM group (12.8 [11.6–14.0] % and 5.5 [4.77–6.28] %, respectively) (Table 1). Likewise, participants in the SM group had increased aPRs for depressive symptoms and suicidal ideation compared to the reference group (no risk factor and NSM group) in all models (Supplementary Figure S1 and S2).

**Table 1:** Characteristics of participants aged 18–25y in the full analyzed sample and according to sexual orientation (N = 6,337; EpiCov study in 2022; not imputed)

Characteristic	Full sample, N = 6,337^1^	95% CI^2^	NSM group, N = 5,594^1^	95% CI^2^	SM group, N = 743^1^	95% CI^2^	p-value^3^
Depressive symptoms during last 15 days							< 0.001
Yes	15.1% (958)	14.0%, 16.3%	12.8% (697)	11.6%, 14.0%	33.9% (261)	29.6%, 38.4%	
Suicidal ideation during last 12 months							< 0.001
Yes	7.4% (511)	6.65%, 8.23%	5.5% (318)	4.77%, 6.28%	23.0% (193)	19.5%, 26.8%	
Sex							< 0.001
Female	45.6% (3,149)	43.9%, 47.3%	44.1% (2,693)	42.3%, 46.0%	57.0% (456)	51.7%, 62.1%	
Age category							0.738
18–21 y	46.2% (2,757)	44.5%, 47.9%	46.3% (2,436)	44.5%, 48.1%	45.4% (321)	40.5%, 50.4%	
22–25 y	53.8% (3,580)	52.1%, 55.5%	53.7% (3,158)	51.9%, 55.5%	54.6% (422)	49.6%, 59.5%	
Educational attainment higher than high school							0.986
Yes	38.4% (2,663)	36.8%, 39.9%	38.4% (2,356)	36.7%, 40.0%	38.3% (307)	33.9%, 43.0%	
Being employed							< 0.001
Yes	29.0% (1,655)	27.5%, 30.6%	30.0% (1,514)	28.3%, 31.7%	21.5% (141)	17.8%, 25.7%	
Perceived financial difficulties							0.155
Yes	12.1% (681)	10.9%, 13.3%	11.8% (584)	10.6%, 13.1%	14.3% (97)	11.2%, 18.1%	
In a relationship							0.603
Yes	25.4% (1,772)	24.1%, 26.8%	25.5% (1,576)	24.1%, 27.0%	24.4% (196)	20.8%, 28.5%	
Living alone							0.004
Yes	26.9% (1,804)	25.5%, 28.4%	26.2% (1,558)	24.7%, 27.7%	33.0% (246)	28.6%, 37.7%	
Urban density							0.683
Rural	23.4% (1,552)	22.0%, 24.8%	23.5% (1,396)	22.1%, 25.1%	22.3% (156)	18.3%, 27.0%	
Intermediate	60.2% (3,894)	58.6%, 61.9%	60.3% (3,412)	58.5%, 62.0%	59.7% (482)	54.6%, 64.7%	
High—Paris area	16.4% (891)	15.1%, 17.8%	16.2% (786)	14.8%, 17.6%	17.9% (105)	13.9%, 22.9%	
Experience of discrimination in the last 5 y							< 0.001
Yes	20.9% (1,346)	19.6%, 22.3%	19.5% (1,089)	18.1%, 20.9%	32.7% (257)	28.5%, 37.2%	
Chronic somatic or mental conditions							< 0.001
Yes	24.6% (1,539)	23.2%, 26.1%	23.6% (1,292)	22.2%, 25.2%	32.7% (247)	28.2%, 37.6%	
History of mental disorders diagnosis							< 0.001
Yes	7.5% (533)	6.76%, 8.40%	6.0% (365)	5.23%, 6.78%	20.2% (168)	16.7%, 24.3%	
Web questionnaire type							0.016
Yes	70.8% (4,592)	69.2%, 72.3%	70.1% (4,011)	68.5%, 71.7%	76.1% (581)	71.6%, 80.1%	

^1^ Weighted % (n (unweighted))

^2^ CI = Confidence Interval

^3^ chi-squared test with Rao & Scott's second-order correction (NSM vs SM)

Sex, educational attainment, being employed, perceived financial difficulties, living alone, experience of discrimination, chronic somatic and mental conditions, history of mental disorders diagnosis contained 545, 2, 1, 24, 8, 9, 76, and 2 missing values, respectively

NSM: Not belonging to Sexual Minority, SM: Sexual Minority

### Moderating effects of sexual orientation on the associations between social factors, depressive symptoms, and suicidal ideation

For depressive symptoms, significant negative interactions (observed joint effect is smaller than expected) were observed between sexual orientation and female sex (IR 0.64 [0.46–0.91]) and 22–25 y age category (IR 0.62 [0.44–0.86]) (Fig. 2). Accordingly, in the Poisson regression model stratified by sexual orientation, female individuals in the NSM group had a higher prevalence of depressive symptoms than their male counterpart (aPR: 1.58 [1.28–1.95]), whereas no significant difference in prevalence was observed between sexes in the SM group (PR: 1.03 [0.78–1.36]) (Fig. 2). Likewise, individuals in the NSM group aged 22–25 y had a higher prevalence of depressive symptoms than their 18–21 y counterparts (PR: 1.32 [1.05–1.67]), whereas no significant difference in prevalence was observed between the age groups in the SM group (0.78 [0.59–1.03]). Regarding suicidal ideation, we observed significant negative interactions (observed joint effect is smaller than expected) between sexual orientation and not being in relationship (IR 0.52 [0.33–0.81]) (Fig. 3). In the Poisson regression stratified by sexual orientation, individuals not being in a relationship in the NSM group had a higher prevalence of suicidal ideation than those being in a relationship (aPR: 1.55 [1.14–2.12]), whereas no significant difference in prevalence was observed according to relationship status in the SM group (aPR 0.82 [0.59–1.13]) (Fig. 3). No positive interactions were detected.

## Sensitivity analyses

In the sensitivity analyses, which adjusted for chronic health conditions and a history of diagnosed mental disorders, as well as for those excluding participants who did not wish to report their sexual orientation, similar results to those from the main analyses were obtained. Nonetheless, some interactions that were borderline significant in the main analyses became significant in the sensitivity analyses (**Supplementary Figure S5, S6, S9, and S10**).

## Discussion

In line with previous findings, in this recent French population-based study, young adults defining themselves as lesbian, gay, bisexual, other, or not defining themselves by their sexual orientation were more likely to suffer from depressive symptoms and suicidal ideation than youth identifying as heterosexual or preferring not to answer. However, some known social risk factors for mental health were found to be associated with depressive symptoms or suicidal ideation only in young adults who identified as heterosexual or preferring not to answer, thus not explaining the higher risk observed in lesbian, gay, bisexual, and not self-defining young adults. Therefore, depression and suicidal ideation may be explained by specific underlying mechanisms in these young adults.

Our results suggest that participants with female sex (vs male participants) and those aged 22–25 y in 2022 (vs those aged 18–21 y) had a higher prevalence of depressive symptoms only in the NSM group, not in the SM group. To the best of our knowledge, no study has explored the moderating effects of sexual orientation on the associations between sex and depression. However, various studies have explored the moderating effects of sex on the associations between sexual orientation and mental health, with inconsistent results. A French study with a nationally representative sample reported that the risk of depression associated with having same-sex partners is higher in men than in women [33]. Another French national survey indicated that the association between being homosexual and depressive symptoms may be greater in women than in men, and that the association between being bisexual and depressive symptoms was smaller in women than in men [34]. However, a nationally representative Swedish study examining associations between sexual orientation and depression diagnosis by gender observed that being homosexual had a smaller association with lifetime diagnostic of depression in women than in men, while being bisexual had greater association in women than in men [45]. With respect to age, a prior study in a sample from the general population reported a U-shaped relationship between psychological well-being and age, with the lowest levels of well-being around ages 45–54y, which is consistent with our results observed in the NSM group [46]. Although this association was not observed in the SM group in our study, a systematic review on the prevalence of major depressive disorders among LGBTQI + people, including 48 articles comprising 4,616,903 individuals, suggested that age was positively associated with a higher prevalence [47]. Our results need to be confirmed by future studies, given that we identified no study investigating the moderating effects of sexual orientation on the association between age and depressive symptoms in young adults. Regarding suicidal ideation, our results suggested that single young adults had a higher prevalence of suicidal ideation than those in a relationship in the NSM group, but not in the SM group. Although we observed no study on the moderating effects of sexual orientation on the association between relationship status and suicidal ideation, a study with a sample of 232 college students from the Philippines reported that being in a relationship may be a risk factor for anxiety and depression among non-heterosexual students, while it was a protective factor among heterosexual students [48]. Our results suggest that being single may not be a risk factor for suicidal ideation among people who define themselves as lesbian, gay, bisexual, other, or not defining themselves by their sexuality. Indeed, disclosure of a sexual minority could be a stressful event, which impact negatively on their mental health [5, 49]. Overall, the modification effects of sexual orientation on the associations between social factors and depressive symptoms/suicidal ideation depend on the study samples, studied variables, period, and how variables are modeled [24–30]. Along with the fact that we identified no study on the moderating effects of sexual orientation observed in the present study, our findings need to be confirmed by future studies using similar study samples and social factors.

In the sensitivity analyses with additional adjustment for chronic health conditions and history of diagnosed mental disorders, we additionally observed moderating effects of sexual orientation on employment status, living in high-population density areas, and discrimination. This may be attributed to the fact that aPR values decreased only in the SM group but not in the NSM group in the sensitivity analyses. Thus, chronic health conditions and a history of mental disorders diagnosis could have important effects on the relationship between these social factors and depressive symptoms and/or suicidal ideation in the SM group, but not in the NSM group. Regarding participants’ experience of discrimination, while those who experienced discrimination had a higher prevalence of depressive symptoms and suicidal ideation than those who did not experience discrimination in both the NSM and the SM groups, the associations between discrimination and outcomes were smaller in the SM group compared to the NSM group. Prior studies have indeed reported that the effects of victimization, bullying, or discrimination for depressive symptoms and suicidal ideation/thoughts were smaller in those belonging to the sexual minority community than who do not in samples of adolescents, high school, and university students [27, 28, 50, 51], which is consistent with our findings. Regarding the sensitivity analyses excluding participants who did not wish to report their sexual orientation, although results for employment status (depressive symptoms) and for perceived financial difficulties (suicidal ideation) became statistically significant, the values of IR were similar to those of main analyses. Furthermore, we observed no other statistical change from main analyses, which indicates that there are no statistical differences in modification effect of sexual orientation between the sensitivity and main analyses.

Although participants belonging to the SM group were more likely to report depressive symptoms and suicidal ideation than those in the NSM group, we observed no social factors having a greater association with depressive symptoms or suicidal ideation in the SM group than in the NSM group. Therefore, unmeasured factors that could not be explored in this study, may mainly contribute to the elevated prevalence of depressive symptoms and suicidal ideation, as described in the minority stress theory [4, 5]. Indeed, prior studies have reported higher levels of internalized stigma, stigma awareness, perceived stigma, and lower levels of perceived safety in sexual minority populations than in their heterosexual counterparts [49]. Concealment and disclosure of sexuality may also have harmful effects on their mental health [5, 49]. In addition, because of the long history of labelling ‘homosexuality’ in international and national disease classification frameworks, which can be stigmatizing, people belonging to sexual minorities are less likely to seek help because of the potential negative attitudes of health service providers [52]. Thus, they may be less likely to receive psychiatric treatment*.* Furthermore, although same-sex marriage has been permitted since 2013 in France, structural stigma, defined as cultural norms and institutional policies at the social level that constrain opportunities, resources, and well-being, may still be present in today’s society [6, 49]. Finally, a new field is emerging in twin and sibling studies using Cholesky's ACE model, which investigates childhood factors (genetic, shared environmental, and non-shared environmental factors) in relation to sexual orientation, depressive symptoms, and suicide attempts [53, 54]. These studies suggested that the increased risk of depression associated with same-sex marriage was partially due to genetic factors among women [54] and that the covariance of genetic factors between sexual orientation and depressive symptoms was greatest at high levels of childhood gender nonconformity, whereas that of environmental effects was greatest at low levels of childhood gender nonconformity [53].

## Limitations and strengths

The limitations of the present study should be noted. First, the question on sexual orientation in the EpiCov cohort was only asked to adult participants not living with a partner, and we decided not to use information on the sex of partner to avoid misclassification bias. Therefore, the results of the present study cannot be generalized to young people living with a partner. However, it should be noted that 81.6% of EpiCov participants aged 18–25y were included in the present analyses. Second, the cross-sectional study design prevents causal inference. Future longitudinal studies could elucidate the chronology of the associations. Third, categorization of continuous variables (e.g., PHQ-9 score, age) may lead to information loss. Finally, because of a methodological constraint regarding number of participants, we grouped diverse categories of sexual orientation in a single group (i.e., SM group), which may oversimplify their diverse experiences. Despite these limitations, this study has several important strengths. To our knowledge, this is the first large-scale epidemiological study to investigate the moderating effects of sexual orientation on the associations between on various social factors, depressive symptoms, and suicidal ideation in young adults. Data were collected in 2022; thus, the results represent the recent situation in France. The observed moderating effects and associations were controlled for the main sociodemographic and health-related risk factors of depression and suicidality. Finally, EpiCov participants were randomly selected from a national tax database covering more than 96% of the general population living in France, and data were weighted to account for non-response, thus reducing potential selection bias.

## Conclusions

This study suggests that in 2022, young adults who identified as lesbian, gay, bisexual, other, or not defining themselves by their sexual orientation are still more likely to experience depressive symptoms and suicidal ideation than their counterparts. However, well-known social risk factors for mental health conditions do not appear to explain this higher prevalence. This work advocates for continuously assessing factors related to mental health according to sexual orientation, as well-known factors can become irrelevant in an ever-evolving context. New generations of young adults might face different types of adversity than their predecessors, some of which affect the mental health of all people and others of which affect the mental health of specific subgroups. Keeping up with these shared and specific factors is necessary to implement effective and inclusive mental health policies.

## Supplementary Information

Below is the link to the electronic supplementary material.

Supplementary Figure S1: Preliminary analysis: multiplicative interactions between sexual orientation and social factors for depressive symptoms in individual model (N= 6,337 aged 18–25y; EpiCov study in 2022; n case/N total contain missing values; weighted and pooled)

Supplementary Figure S2: Preliminary analysis: multiplicative interactions between sexual orientation and social factors for suicidal ideation in individual model (N= 6,337 aged 18–25y; EpiCov study in 2022; n case/N total contain missing values; weighted and pooled)

Supplementary Figure S3: Preliminary and sensitivity analysis: multiplicative interactions between sexual orientation and social factors for depressive symptoms in individual model (N= 6,337 aged 18–25y; EpiCov study in 2022; n case/N total contain missing values; weighted and pooled; additional adjustment on chronic health conditions and a history of mental disorders diagnosis)

Supplementary Figure S4: Preliminary and sensitivity analysis: multiplicative interactions between sexual orientation and social factors for suicidal ideation in individual model (N= 6,337 aged 18–25y; EpiCov study; in 2022; n case/N total contain missing values; weighted and pooled; additional adjustment on chronic health conditions a history of mental disorders diagnosis)

Supplementary Figure S5. Sensitivity analysis: associations between social factors and depressive symptoms according to sexual orientation (N= 6,337 aged 18–25y; EpiCov study in 2022; weighted and pooled; additional adjustment on chronic health conditions and a history of mental disorders diagnosis)

Supplementary Figure S6. Sensitivity analysis: associations between social factors and suicidal ideation according to sexual orientation (N= 6,337 aged 18–25y; EpiCov study in 2022; weighted and pooled; additional adjustment on chronic health conditions and a history of mental disorders diagnosis)

Supplementary Figure S7: Preliminary and sensitivity analysis: multiplicative interactions between sexual orientation and social factors for depressive symptoms in individual model (N= 5,544 aged 18–25y; EpiCov study in 2022; n case/N total contain missing values; weighted and pooled; exclusion of participants who did not wish to report their sexual orientation)

Supplementary Figure S8: Preliminary and sensitivity analysis: multiplicative interactions between sexual orientation and social factors for suicidal ideation in individual model (N= 5,544 aged 18–25y; EpiCov study; in 2022; n case/N total contain missing values; weighted and pooled; exclusion of participants who did not wish to report their sexual orientation)

Supplementary Figure S9. Sensitivity analysis: associations between social factors and depressive symptoms according to sexual orientation (N= 5,544 aged 18–25y; EpiCov study in 2022; weighted and pooled; exclusion of participants who did not wish to report their sexual orientation)

Supplementary Figure S10. Sensitivity analysis: associations between social factors and suicidal ideation according to sexual orientation (N= 5,544 aged 18–25y; EpiCov study in 2022; weighted and pooled; exclusion of participants who did not wish to report their sexual orientation)

Supplementary file11 (DOCX 43 kb)

## Data Availability

The non-aggregated individual data cannot be shared publicly because of European Regulation 2016/679. Nonetheless, these data can be made available after submission to approval of French Ethics and Regulatory Committee procedure (Comité du Secret Statistique, CESREES and CNIL). The EpiCov dataset is available for research purposes on CASD (https://www.casd.eu/).

## Abbreviations

CATIs: Computer-Assisted Telephone Interviews
CAWIs: Self-Computer-Assisted Web Interviews
EpiCov: *Epidemiology et Conditions de vie sous le* COVID-19
ICD: International Classification of Diseases
IR: Interaction Ratio
INSEE: National Institute for Statistics and Economic Studies
LGBTQI +: Lesbian, Gay, Bisexual, Transgender, Queer, Intersex, and inclusive of individuals who identify as part of sexual and gender diverse communities
NSM: Not belonging to Sexual Minority
PHQ-9: 9 Items of the Patient Health Questionnaire
PR: Prevalence Ratio
SM: Sexual Minority
