# Supplementary material for: Moderating effects of self-defined sexual orientation on the relation between social factors and depressive symptoms or suicidal ideation among French young adults
Source: Soc Psychiatry Psychiatr Epidemiol. 2025 Jun 23;60(10):2455–68. doi: 10.1007/s00127-025-02951-y (PMC12449324; doi:10.1007/s00127-025-02951-y)
Supplement: Supplementary file 1 — Supplementary Figure S1: Preliminary analysis: multiplicative interactions between sexual orientation and social factors for depressive symptoms in individual model (N= 6,337 aged 18–25y; EpiCov study in 2022; n case/N total contain missing values; weighted and pooled) [file 127_2025_2951_MOESM1_ESM.pdf]

Factor	n case/N total	.	PR(CI95%)	p value
Sex at birth		IR	0.69 (0.49 − 0.96)	0.030
Male:NSM	194/2401		1.00	
Female:NSM	434/2693		1.58 (1.28 − 1.94)	
Male:SM	75/242		2.63 (1.97 − 3.50)	
Female:SM	169/456		2.85 (2.25 − 3.62)	
Age category		IR	0.64 (0.47 − 0.89)	0.007
18 − 21 y:NSM	378/3158		1.00	
22 − 25 y:NSM	319/2436		1.30 (1.04 − 1.62)	
18 − 21 y:SM	163/422		2.50 (2.03 − 3.08)	
22 − 25 y:SM	98/321		2.10 (1.63 − 2.70)	
Educational attainment		IR	1.21 (0.89 − 1.66)	0.229
Higher than bac:NSM	297/2356		1.00	
Bac and lower:NSM	400/3236		1.06 (0.86 − 1.31)	
Higher than bac:SM	96/307		1.83 (1.43 − 2.34)	
Bac and lower:SM	165/436		2.35 (1.87 − 2.94)	
Employment status		IR	0.76 (0.50 − 1.16)	0.209
Being employed:NSM	135/1514		1.00	
Not being employed:NSM	562/4079		1.60 (1.23 − 2.09)	
Being employed:SM	38/141		2.59 (1.76 − 3.83)	
Not being employed:SM	223/602		3.17 (2.41 − 4.19)	
Perceived financial difficulties		IR	0.85 (0.59 − 1.23)	0.393
No:NSM	553/4990		1.00	
Yes:NSM	143/584		1.74 (1.39 − 2.18)	
No:SM	203/642		2.15 (1.79 − 2.58)	
Yes:SM	56/97		3.18 (2.40 − 4.23)	
In relationship		IR	0.97 (0.69 − 1.37)	0.862
Yes:NSM	208/1576		1.00	
No:NSM	489/4018		1.03 (0.84 − 1.27)	
Yes:SM	75/196		2.12 (1.58 − 2.84)	
No:SM	186/547		2.12 (1.69 − 2.66)	
Living alone		IR	0.74 (0.54 − 1.02)	0.068
No:NSM	452/4030		1.00	
Yes:NSM	244/1558		1.38 (1.13 − 1.69)	
No:SM	178/495		2.29 (1.88 − 2.79)	
Yes:SM	82/246		2.35 (1.83 − 3.01)	
Urban density		IR	1.06 (0.72 − 1.56)	0.770
Rural:NSM	159/1396		1.00	
Intermediate:NSM	438/3412		0.98 (0.78 − 1.23)	
Rural:SM	53/156		2.01 (1.43 − 2.84)	
Intermediate:SM	168/482		2.08 (1.61 − 2.70)	
Urban density		IR	0.94 (0.56 − 1.59)	0.825
Rural:NSM	159/1396		1.00	
High−Paris:NSM	100/786		1.08 (0.79 − 1.49)	
Rural:SM	53/156		2.08 (1.47 − 2.93)	
High−Paris:SM	40/105		2.12 (1.47 − 3.05)	
Discrimination		IR	0.75 (0.55 − 1.02)	0.068
No:NSM	417/4496		1.00	
Yes:NSM	279/1089		2.46 (2.02 − 2.99)	
No:SM	128/486		2.39 (1.89 − 3.03)	
Yes:SM	133/257		4.40 (3.58 − 5.40)	

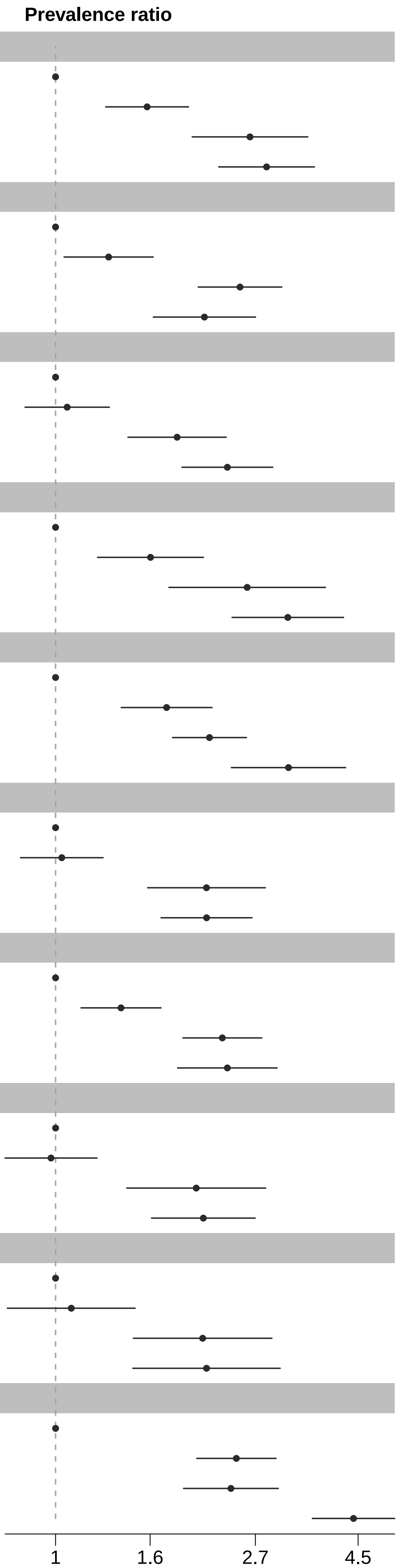

PR: Prevalence ratio, CI: Confidence interaval,IR: Interaction ratio
NSM: Not belonging to sexual minority, SM: Sexual minority
