# Supplementary material for: Moderating effects of self-defined sexual orientation on the relation between social factors and depressive symptoms or suicidal ideation among French young adults
Source: Soc Psychiatry Psychiatr Epidemiol. 2025 Jun 23;60(10):2455–68. doi: 10.1007/s00127-025-02951-y (PMC12449324; doi:10.1007/s00127-025-02951-y)
Supplement: Supplementary file 2 — Supplementary Figure S2: Preliminary analysis: multiplicative interactions between sexual orientation and social factors for suicidal ideation in individual model (N= 6,337 aged 18–25y; EpiCov study in 2022; n case/N total contain missing values; weighted and pooled) [file 127_2025_2951_MOESM2_ESM.pdf]

Factor	n case/N total	.	PR(CI95%)	p value
Sex at birth		IR	0.85 (0.53 − 1.37)	0.514
Male:NSM	107/2401		1.00	
Female:NSM	190/2693		1.38 (1.03 − 1.86)	
Male:SM	56/242		3.62 (2.45 − 5.34)	
Female:SM	124/456		4.28 (3.15 − 5.81)	
Age category		IR	0.82 (0.54 − 1.26)	0.369
18 − 21 y:NSM	169/3158		1.00	
22 − 25 y:NSM	149/2436		1.33 (0.97 − 1.84)	
18 − 21 y:SM	108/422		3.59 (2.71 − 4.75)	
22 − 25 y:SM	85/321		3.93 (2.86 − 5.41)	
Educational attainment		IR	0.92 (0.61 − 1.39)	0.685
Higher than bac:NSM	132/2356		1.00	
Bac and lower:NSM	186/3236		1.26 (0.94 − 1.69)	
Higher than bac:SM	74/307		3.47 (2.51 − 4.79)	
Bac and lower:SM	119/436		4.00 (2.96 − 5.39)	
Employment status		IR	0.63 (0.36 − 1.08)	0.092
Being employed:NSM	62/1514		1.00	
Not being employed:NSM	256/4079		1.87 (1.29 − 2.70)	
Being employed:SM	31/141		4.83 (2.95 − 7.91)	
Not being employed:SM	162/602		5.67 (3.90 − 8.24)	
Perceived financial difficulties		IR	0.58 (0.34 − 0.99)	0.046
No:NSM	257/4990		1.00	
Yes:NSM	61/584		1.59 (1.12 − 2.25)	
No:SM	161/642		3.63 (2.86 − 4.61)	
Yes:SM	31/97		3.34 (2.22 − 5.03)	
In relationship		IR	0.57 (0.36 − 0.89)	0.015
Yes:NSM	71/1576		1.00	
No:NSM	247/4018		1.55 (1.14 − 2.11)	
Yes:SM	58/196		5.01 (3.40 − 7.38)	
No:SM	135/547		4.44 (3.18 − 6.21)	
Living alone		IR	0.70 (0.44 − 1.10)	0.125
No:NSM	206/4030		1.00	
Yes:NSM	111/1558		1.49 (1.10 − 2.02)	
No:SM	132/495		3.70 (2.85 − 4.79)	
Yes:SM	61/246		3.85 (2.77 − 5.37)	
Urban density		IR	0.88 (0.53 − 1.48)	0.631
Rural:NSM	61/1396		1.00	
Intermediate:NSM	205/3412		1.20 (0.84 − 1.72)	
Rural:SM	42/156		3.89 (2.47 − 6.11)	
Intermediate:SM	122/482		4.13 (2.81 − 6.05)	
Urban density		IR	0.69 (0.35 − 1.34)	0.270
Rural:NSM	61/1396		1.00	
High−Paris:NSM	52/786		1.35 (0.88 − 2.09)	
Rural:SM	42/156		3.49 (2.27 − 5.37)	
High−Paris:SM	29/105		3.25 (1.95 − 5.39)	
Discrimination		IR	0.72 (0.48 − 1.10)	0.129
No:NSM	179/4496		1.00	
Yes:NSM	137/1089		2.75 (2.08 − 3.64)	
No:SM	97/486		3.85 (2.81 − 5.26)	
Yes:SM	96/257		7.66 (5.74 − 10.23)	

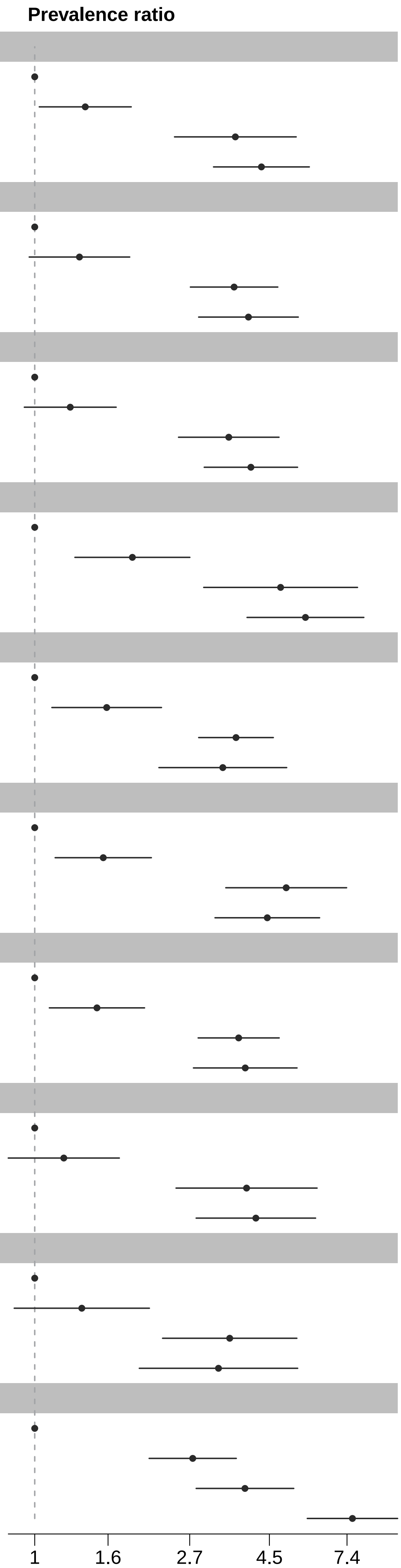

PR: Prevalence ratio, CI: Confidence interaval,IR: Interaction ratio

NSM: Not belonging to sexual minority, SM: Sexual minority
