# Supplementary material for: Moderating effects of self-defined sexual orientation on the relation between social factors and depressive symptoms or suicidal ideation among French young adults
Source: Soc Psychiatry Psychiatr Epidemiol. 2025 Jun 23;60(10):2455–68. doi: 10.1007/s00127-025-02951-y (PMC12449324; doi:10.1007/s00127-025-02951-y)
Supplement: Supplementary file 3 — Supplementary Figure S3: Preliminary and sensitivity analysis: multiplicative interactions between sexual orientation and social factors for depressive symptoms in individual model (N= 6,337 aged 18–25y; EpiCov study in 2022; n case/N total contain missing values; weighted and pooled; additional adjustment on chronic health conditions and a history of mental disorders diagnosis) [file 127_2025_2951_MOESM3_ESM.pdf]

Factor	n case/N total	.	PR(CI95%)	p value
Sex at birth		IR	0.70 (0.50 − 0.98)	0.036
Male:NSM	194/2401		1.00	
Female:NSM	434/2693		1.53 (1.25 − 1.87)	
Male:SM	75/242		2.21 (1.65 − 2.95)	
Female:SM	169/456		2.37 (1.87 − 2.99)	
Age category		IR	0.60 (0.44 − 0.82)	0.001
18 − 21 y:NSM	378/3158		1.00	
22 − 25 y:NSM	319/2436		1.20 (0.96 − 1.50)	
18 − 21 y:SM	163/422		2.19 (1.80 − 2.67)	
22 − 25 y:SM	98/321		1.57 (1.20 − 2.05)	
Educational attainment		IR	1.17 (0.86 − 1.58)	0.313
Higher than bac:NSM	297/2356		1.00	
Bac and lower:NSM	400/3236		1.02 (0.83 − 1.25)	
Higher than bac:SM	96/307		1.59 (1.25 − 2.02)	
Bac and lower:SM	165/436		1.89 (1.50 − 2.38)	
Employment status		IR	0.68 (0.45 − 1.04)	0.075
Being employed:NSM	135/1514		1.00	
Not being employed:NSM	562/4079		1.54 (1.19 − 2.00)	
Being employed:SM	38/141		2.42 (1.63 − 3.58)	
Not being employed:SM	223/602		2.54 (1.93 − 3.35)	
Perceived financial difficulties		IR	0.93 (0.65 − 1.32)	0.673
No:NSM	553/4990		1.00	
Yes:NSM	143/584		1.55 (1.23 − 1.96)	
No:SM	203/642		1.79 (1.49 − 2.15)	
Yes:SM	56/97		2.57 (1.93 − 3.43)	
In relationship		IR	0.97 (0.70 − 1.34)	0.837
Yes:NSM	208/1576		1.00	
No:NSM	489/4018		1.04 (0.85 − 1.27)	
Yes:SM	75/196		1.80 (1.37 − 2.37)	
No:SM	186/547		1.81 (1.44 − 2.27)	
Living alone		IR	0.74 (0.54 − 1.02)	0.064
No:NSM	452/4030		1.00	
Yes:NSM	244/1558		1.33 (1.09 − 1.63)	
No:SM	178/495		1.95 (1.60 − 2.37)	
Yes:SM	82/246		1.92 (1.50 − 2.46)	
Urban density		IR	0.97 (0.66 − 1.42)	0.861
Rural:NSM	159/1396		1.00	
Intermediate:NSM	438/3412		0.99 (0.79 − 1.25)	
Rural:SM	53/156		1.85 (1.31 − 2.62)	
Intermediate:SM	168/482		1.77 (1.38 − 2.29)	
Urban density		IR	0.81 (0.49 − 1.35)	0.417
Rural:NSM	159/1396		1.00	
High−Paris:NSM	100/786		1.12 (0.82 − 1.53)	
Rural:SM	53/156		1.95 (1.38 − 2.76)	
High−Paris:SM	40/105		1.77 (1.21 − 2.60)	
Discrimination		IR	0.69 (0.51 − 0.93)	0.016
No:NSM	417/4496		1.00	
Yes:NSM	279/1089		2.19 (1.80 − 2.67)	
No:SM	128/486		2.11 (1.68 − 2.66)	
Yes:SM	133/257		3.19 (2.58 − 3.95)	

Prevalence ratio

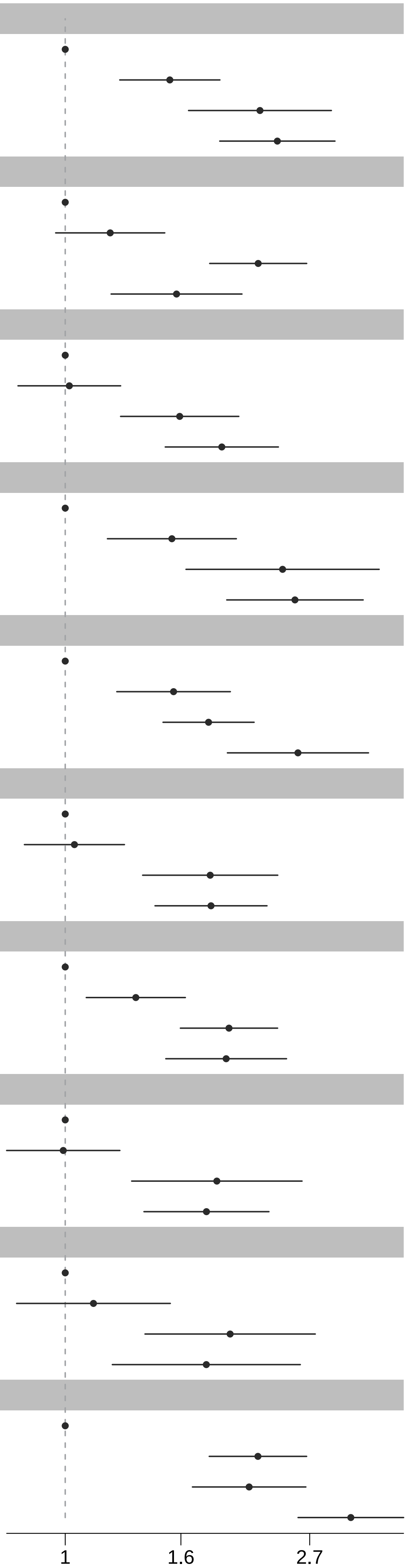

PR: Prevalence ratio, CI: Confidence interaval,IR: Interaction ratio
NSM: Not belonging to sexual minority, SM: Sexual minority
