# Supplementary figures and images for: Moderating effects of self-defined sexual orientation on the relation between social factors and depressive symptoms or suicidal ideation among French young adults
Source: Soc Psychiatry Psychiatr Epidemiol. 2025 Jun 23;60(10):2455–68. doi: 10.1007/s00127-025-02951-y (PMC12449324; doi:10.1007/s00127-025-02951-y)

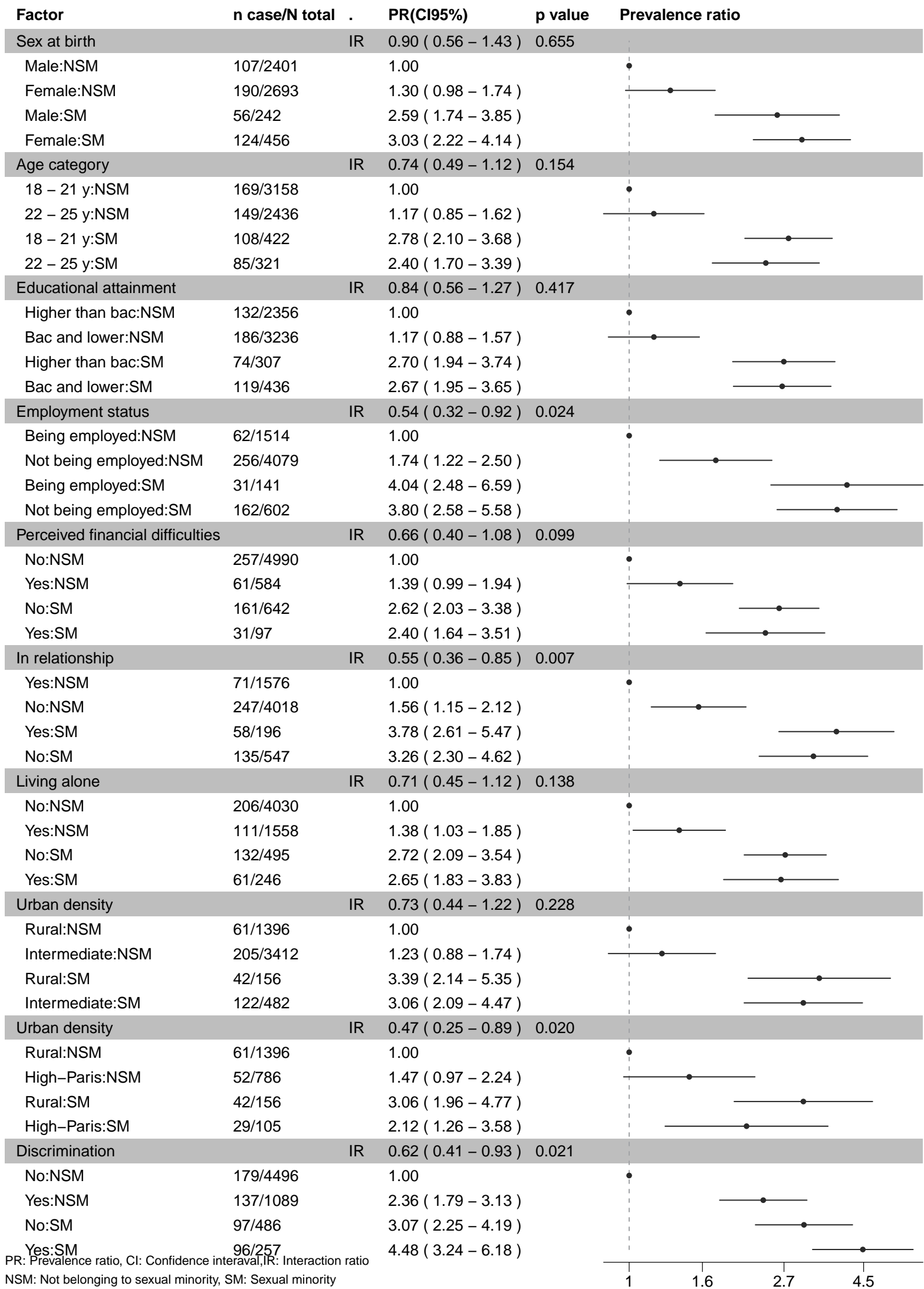

Supplement: Supplementary file 4 — Supplementary Figure S4: Preliminary and sensitivity analysis: multiplicative interactions between sexual orientation and social factors for suicidal ideation in individual model (N= 6,337 aged 18–25y; EpiCov study; in 2022; n case/N total contain missing values; weighted and pooled; additional adjustment on chronic health conditions a history of mental disorders diagnosis) [file 127_2025_2951_MOESM4_ESM.pdf]
