# Supplementary material for: Moderating effects of self-defined sexual orientation on the relation between social factors and depressive symptoms or suicidal ideation among French young adults
Source: Soc Psychiatry Psychiatr Epidemiol. 2025 Jun 23;60(10):2455–68. doi: 10.1007/s00127-025-02951-y (PMC12449324; doi:10.1007/s00127-025-02951-y)
Supplement: Supplementary file 5 — Supplementary Figure S5. Sensitivity analysis: associations between social factors and depressive symptoms according to sexual orientation (N= 6,337 aged 18–25y; EpiCov study in 2022; weighted and pooled; additional adjustment on chronic health conditions and a history of mental disorders diagnosis) [file 127_2025_2951_MOESM5_ESM.pdf]

Factor	n case/N total	.	PR(CI95%)	p value
Sex at birth		IR	0.68 (0.49 − 0.95)	0.024
Male:NSM	194/2401		1.00	
Female:NSM	434/2693		1.53 (1.24 − 1.88)	<0.001
Male:SM	75/242		1.00	
Female:SM	169/456		1.04 (0.81 − 1.35)	0.756
Age category		IR	0.53 (0.39 − 0.74)	<0.001
18 − 21 y:NSM	378/3158		1.00	
22 − 25 y:NSM	319/2436		1.23 (0.97 − 1.56)	0.084
18 − 21 y:SM	163/422		1.00	
22 − 25 y:SM	98/321		0.66 (0.51 − 0.87)	0.003
Educational attainment		IR		NS
Higher than bac:NSM	297/2356		1.00	
Bac and lower:NSM	400/3236		1.08 (0.87 − 1.35)	0.478
Higher than bac:SM	96/307		1.00	
Bac and lower:SM	165/436		1.01 (0.77 − 1.32)	0.934
Employment status		IR	0.58 (0.37 − 0.90)	0.015
Being employed:NSM	135/1514		1.00	
Not being employed:NSM	562/4079		1.61 (1.23 − 2.10)	<0.001
Being employed:SM	38/141		1.00	
Not being employed:SM	223/602		0.99 (0.69 − 1.40)	0.935
Perceived fincial difficulties		IR		NS
No:NSM	553/4990		1.00	
Yes:NSM	143/584		1.49 (1.18 − 1.89)	0.001
No:SM	203/642		1.00	
Yes:SM	56/97		1.54 (1.17 − 2.04)	0.002
In relationship		IR		NS
Yes:NSM	208/1576		1.00	
No:NSM	489/4018		1.06 (0.86 − 1.30)	0.583
Yes:SM	75/196		1.00	
No:SM	186/547		0.96 (0.76 − 1.23)	0.757
Living alone		IR	0.78 (0.56 − 1.08)	0.133
No:NSM	452/4030		1.00	
Yes:NSM	244/1558		1.32 (1.07 − 1.62)	0.009
No:SM	178/495		1.00	
Yes:SM	82/246		1.00 (0.77 − 1.30)	1.000
Urban density		IR		NS
Rural:NSM	159/1396		1.00	
Intermediate:NSM	438/3412		0.97 (0.77 − 1.23)	0.813
Rural:SM	53/156		1.00	
Intermediate:SM	168/482		0.96 (0.71 − 1.28)	0.766
Urban density		IR		NS
Rural:NSM	159/1396		1.00	
High−Paris:NSM	100/786		1.11 (0.81 − 1.52)	0.512
Rural:SM	53/156		1.00	
High−Paris:SM	40/105		0.89 (0.58 − 1.35)	0.580
Discrimination		IR	0.73 (0.54 − 0.99)	0.040
No:NSM	417/4496		1.00	
Yes:NSM	279/1089		2.12 (1.73 − 2.60)	<0.001
No:SM	128/486		1.00	
Yes:SM	133/257		1.54 (1.21 − 1.97)	<0.001

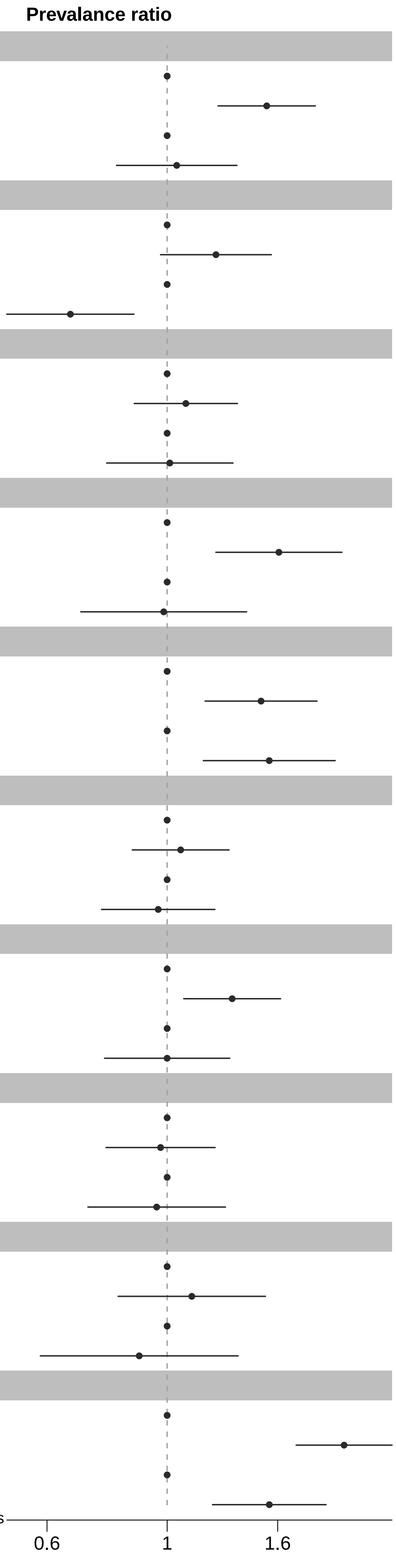

PR: Prevalence ratio, CI: Confidence interaval, NS: Interaction test non significatif in preliminary analysis
NSM: Not belonging to sexual minority, IR: Interaction ratio, SM: Sexual minority
