# Supplementary material for: Moderating effects of self-defined sexual orientation on the relation between social factors and depressive symptoms or suicidal ideation among French young adults
Source: Soc Psychiatry Psychiatr Epidemiol. 2025 Jun 23;60(10):2455–68. doi: 10.1007/s00127-025-02951-y (PMC12449324; doi:10.1007/s00127-025-02951-y)
Supplement: Supplementary file 6 — Supplementary Figure S6. Sensitivity analysis: associations between social factors and suicidal ideation according to sexual orientation (N= 6,337 aged 18–25y; EpiCov study in 2022; weighted and pooled; additional adjustment on chronic health conditions and a history of mental disorders diagnosis) [file 127_2025_2951_MOESM6_ESM.pdf]

Factor	n case/N total	.	PR(CI95%)	p value
Sex at birth		IR		NS
Male:NSM	107/2401		1.00	
Female:NSM	190/2693		1.26 (0.94 − 1.68)	0.120
Male:SM	56/242		1.00	
Female:SM	124/456		1.15 (0.84 − 1.60)	0.385
Age category		IR	0.75 (0.49 − 1.14)	0.179
18 − 21 y:NSM	169/3158		1.00	
22 − 25 y:NSM	149/2436		1.20 (0.84 − 1.70)	0.309
18 − 21 y:SM	108/422		1.00	
22 − 25 y:SM	85/321		0.87 (0.64 − 1.18)	0.365
Educational attainment		IR		NS
Higher than bac:NSM	132/2356		1.00	
Bac and lower:NSM	186/3236		1.22 (0.88 − 1.67)	0.229
Higher than bac:SM	74/307		1.00	
Bac and lower:SM	119/436		0.92 (0.68 − 1.26)	0.619
Employment status		IR	0.52 (0.30 − 0.91)	0.022
Being employed:NSM	62/1514		1.00	
Not being employed:NSM	256/4079		1.75 (1.20 − 2.56)	0.004
Being employed:SM	31/141		1.00	
Not being employed:SM	162/602		1.01 (0.66 − 1.52)	0.981
Perceived financial difficulties		IR	0.70 (0.43 − 1.14)	0.156
No:NSM	257/4990		1.00	
Yes:NSM	61/584		1.27 (0.89 − 1.80)	0.191
No:SM	161/642		1.00	
Yes:SM	31/97		0.93 (0.65 − 1.32)	0.681
In relationship		IR	0.54 (0.35 − 0.82)	0.004
Yes:NSM	71/1576		1.00	
No:NSM	247/4018		1.55 (1.14 − 2.10)	0.005
Yes:SM	58/196		1.00	
No:SM	135/547		0.82 (0.62 − 1.10)	0.192
Living alone		IR	0.65 (0.41 − 1.02)	0.061
No:NSM	206/4030		1.00	
Yes:NSM	111/1558		1.42 (1.05 − 1.93)	0.023
No:SM	132/495		1.00	
Yes:SM	61/246		0.91 (0.65 − 1.29)	0.603
Urban density		IR		NS
Rural:NSM	61/1396		1.00	
Intermediate:NSM	205/3412		1.20 (0.85 − 1.69)	0.295
Rural:SM	42/156		1.00	
Intermediate:SM	122/482		0.91 (0.63 − 1.31)	0.621
Urban density		IR	0.47 (0.25 − 0.92)	0.027
Rural:NSM	61/1396		1.00	
High−Paris:NSM	52/786		1.53 (0.99 − 2.35)	0.054
Rural:SM	42/156		1.00	
High−Paris:SM	29/105		0.74 (0.45 − 1.20)	0.221
Discrimination		IR	0.64 (0.43 − 0.96)	0.029
No:NSM	179/4496		1.00	
Yes:NSM	137/1089		2.17 (1.63 − 2.89)	<0.001
No:SM	97/486		1.00	
Yes:SM	96/257		1.56 (1.16 − 2.11)	0.004

Prevalance ratio

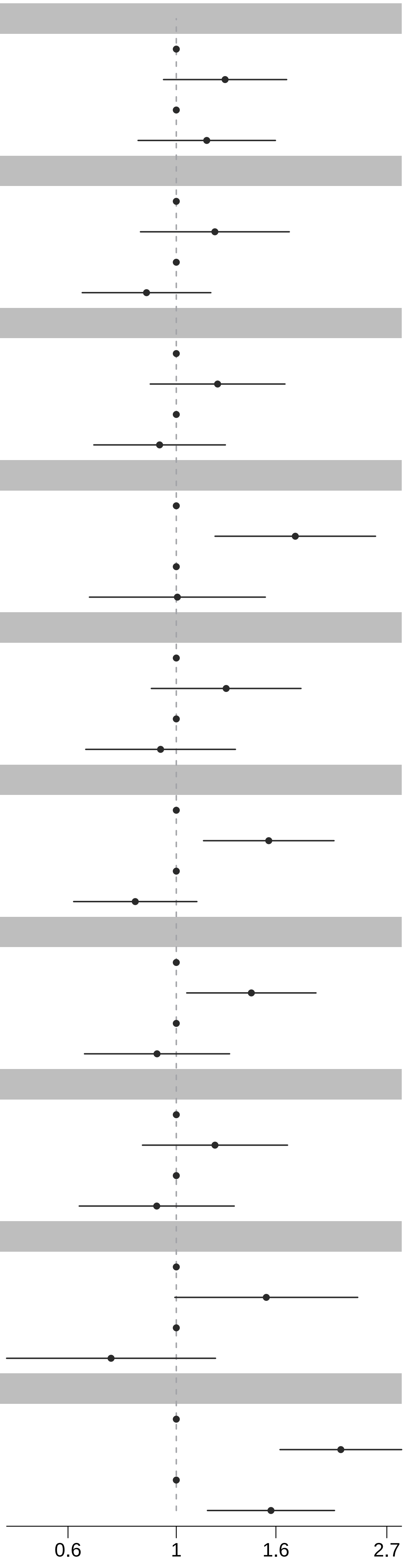

PR: Prevalence ratio, CI: Confidence interaval, NS: Interaction test non significatif in preliminary analysis
NSM: Not belonging to sexual minority, IR: Interaction ratio, SM: Sexual minority
