# Supplementary material for: Moderating effects of self-defined sexual orientation on the relation between social factors and depressive symptoms or suicidal ideation among French young adults
Source: Soc Psychiatry Psychiatr Epidemiol. 2025 Jun 23;60(10):2455–68. doi: 10.1007/s00127-025-02951-y (PMC12449324; doi:10.1007/s00127-025-02951-y)
Supplement: Supplementary file 7 — Supplementary Figure S7: Preliminary and sensitivity analysis: multiplicative interactions between sexual orientation and social factors for depressive symptoms in individual model (N= 5,544 aged 18–25y; EpiCov study in 2022; n case/N total contain missing values; weighted and pooled; exclusion of participants who did not wish to report their sexual orientation) [file 127_2025_2951_MOESM7_ESM.pdf]

Factor	n case/N total	.	PR(CI95%)	p value
Sex at birth		IR	0.71 (0.50 − 1.00)	0.051
Male:NSM	177/2110			
Female:NSM	375/2304		1.50 (1.21 − 1.87)	
Male:SM	75/242		2.47 (1.83 − 3.32)	
Female:SM	169/456		2.61 (2.04 − 3.35)	
Age category		IR	0.64 (0.46 − 0.90)	0.009
18 − 21 y:NSM	324/2684			
22 − 25 y:NSM	283/2117		1.31 (1.05 − 1.64)	
18 − 21 y:SM	163/422		2.39 (1.93 − 2.96)	
22 − 25 y:SM	98/321		2.02 (1.56 − 2.61)	
Educational attainment		IR	1.16 (0.85 − 1.60)	0.353
Higher than bac:NSM	262/2082			
Bac and lower:NSM	345/2718		1.10 (0.89 − 1.37)	
Higher than bac:SM	96/307		1.79 (1.40 − 2.30)	
Bac and lower:SM	165/436		2.29 (1.82 − 2.89)	
Employment status		IR	0.70 (0.45 − 1.08)	0.106
Being employed:NSM	114/1303			
Not being employed:NSM	493/3498		1.73 (1.31 − 2.29)	
Being employed:SM	38/141		2.67 (1.78 − 3.99)	
Not being employed:SM	223/602		3.23 (2.41 − 4.33)	
Perceived financial difficulties		IR	0.79 (0.54 − 1.16)	0.230
No:NSM	484/4329			
Yes:NSM	122/463		1.83 (1.43 − 2.35)	
No:SM	203/642		2.09 (1.73 − 2.52)	
Yes:SM	56/97		3.04 (2.27 − 4.06)	
In relationship		IR	0.96 (0.68 − 1.36)	0.824
Yes:NSM	193/1488			
No:NSM	414/3313		1.05 (0.85 − 1.29)	
Yes:SM	75/196		2.03 (1.51 − 2.73)	
No:SM	186/547		2.05 (1.63 − 2.58)	
Living alone		IR	0.77 (0.56 − 1.08)	0.129
No:NSM	391/3408			
Yes:NSM	215/1388		1.34 (1.08 − 1.65)	
No:SM	178/495		2.15 (1.76 − 2.64)	
Yes:SM	82/246		2.23 (1.73 − 2.87)	
Urban density		IR	1.00 (0.67 − 1.49)	0.996
Rural:NSM	134/1200			
Intermediate:NSM	385/2930		1.02 (0.80 − 1.31)	
Rural:SM	53/156		2.01 (1.41 − 2.87)	
Intermediate:SM	168/482		2.06 (1.56 − 2.71)	
Urban density		IR	0.90 (0.52 − 1.55)	0.700
Rural:NSM	134/1200			
High−Paris:NSM	88/671		1.14 (0.81 − 1.60)	
Rural:SM	53/156		1.97 (1.38 − 2.82)	
High−Paris:SM	40/105		2.05 (1.40 − 3.02)	
Discrimination		IR	0.72 (0.52 − 0.98)	0.040
No:NSM	369/3878			
Yes:NSM	237/920		2.56 (2.07 − 3.16)	
No:SM	128/486		2.33 (1.83 − 2.97)	
Yes:SM	133/257		4.27 (3.45 − 5.29)	

Prevalence ratio

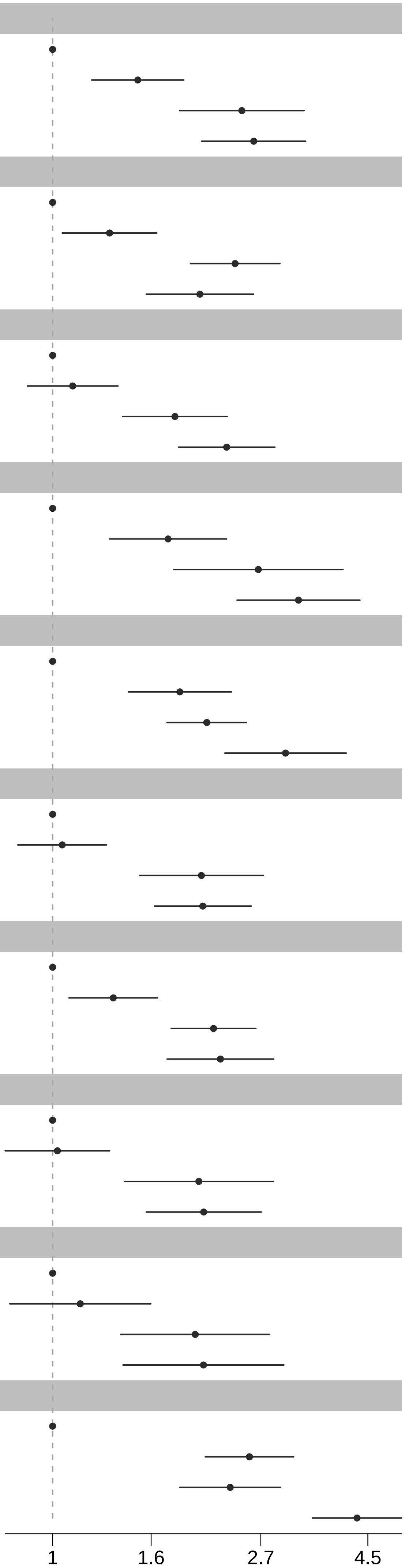

PR: Prevalence ratio, CI: Confidence interaval,IR: Interaction ratio

NSM: Not belonging to sexual minority, SM: Sexual minority
