# Supplementary material for: Moderating effects of self-defined sexual orientation on the relation between social factors and depressive symptoms or suicidal ideation among French young adults
Source: Soc Psychiatry Psychiatr Epidemiol. 2025 Jun 23;60(10):2455–68. doi: 10.1007/s00127-025-02951-y (PMC12449324; doi:10.1007/s00127-025-02951-y)
Supplement: Supplementary file 8 — Supplementary Figure S8: Preliminary and sensitivity analysis: multiplicative interactions between sexual orientation and social factors for suicidal ideation in individual model (N= 5,544 aged 18–25y; EpiCov study; in 2022; n case/N total contain missing values; weighted and pooled; exclusion of participants who did not wish to report their sexual orientation) [file 127_2025_2951_MOESM8_ESM.pdf]

Factor	n case/N total	.	PR(CI95%)	p value
Sex at birth		IR	0.91 (0.57 − 1.47)	0.705
Male:NSM	97/2110			
Female:NSM	161/2304		1.31 (0.96 − 1.79)	
Male:SM	56/242		3.35 (2.25 − 4.99)	
Female:SM	124/456		4.00 (2.91 − 5.49)	
Age category		IR	0.81 (0.52 − 1.25)	0.338
18 − 21 y:NSM	142/2684			
22 − 25 y:NSM	134/2117		1.36 (0.96 − 1.92)	
18 − 21 y:SM	108/422		3.50 (2.61 − 4.70)	
22 − 25 y:SM	85/321		3.83 (2.75 − 5.34)	
Educational attainment		IR	0.91 (0.59 − 1.40)	0.681
Higher than bac:NSM	118/2082			
Bac and lower:NSM	158/2718		1.27 (0.93 − 1.74)	
Higher than bac:SM	74/307		3.36 (2.41 − 4.68)	
Bac and lower:SM	119/436		3.89 (2.85 − 5.31)	
Employment status		IR	0.61 (0.35 − 1.06)	0.080
Being employed:NSM	55/1303			
Not being employed:NSM	221/3498		1.95 (1.32 − 2.88)	
Being employed:SM	31/141		4.80 (2.89 − 7.96)	
Not being employed:SM	162/602		5.67 (3.82 − 8.40)	
Perceived financial difficulties		IR	0.51 (0.30 − 0.88)	0.017
No:NSM	221/4329			
Yes:NSM	55/463		1.79 (1.23 − 2.61)	
No:SM	161/642		3.61 (2.81 − 4.63)	
Yes:SM	31/97		3.30 (2.17 − 5.00)	
In relationship		IR	0.55 (0.35 − 0.87)	0.010
Yes:NSM	66/1488			
No:NSM	210/3313		1.62 (1.18 − 2.23)	
Yes:SM	58/196		4.97 (3.35 − 7.37)	
No:SM	135/547		4.43 (3.15 − 6.24)	
Living alone		IR	0.72 (0.45 − 1.16)	0.179
No:NSM	178/3408			
Yes:NSM	97/1388		1.43 (1.03 − 1.97)	
No:SM	132/495		3.53 (2.69 − 4.64)	
Yes:SM	61/246		3.64 (2.58 − 5.13)	
Urban density		IR	0.81 (0.47 − 1.39)	0.446
Rural:NSM	51/1200			
Intermediate:NSM	184/2930		1.31 (0.88 − 1.93)	
Rural:SM	42/156		3.84 (2.39 − 6.17)	
Intermediate:SM	122/482		4.07 (2.70 − 6.14)	
Urban density		IR	0.69 (0.33 − 1.41)	0.305
Rural:NSM	51/1200			
High−Paris:NSM	41/671		1.32 (0.80 − 2.16)	
Rural:SM	42/156		3.72 (2.33 − 5.95)	
High−Paris:SM	29/105		3.45 (2.04 − 5.85)	
Discrimination		IR	0.73 (0.47 − 1.12)	0.148
No:NSM	158/3878			
Yes:NSM	118/920		2.71 (2.00 − 3.67)	
No:SM	97/486		3.70 (2.68 − 5.11)	
Yes:SM	96/257		7.27 (5.36 − 9.85)	

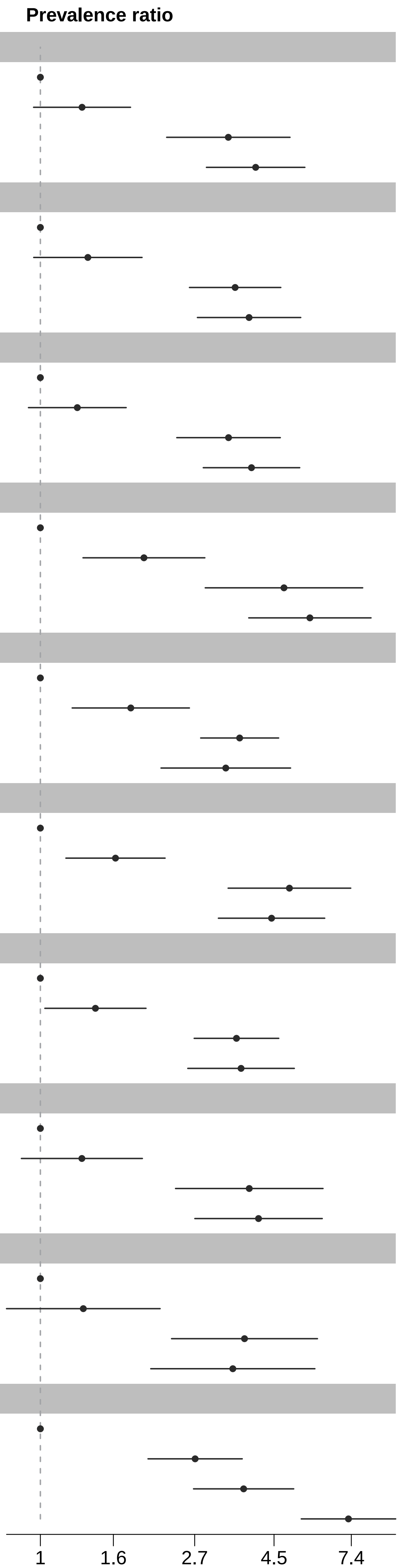

PR: Prevalence ratio, CI: Confidence interaval,IR: Interaction ratio

NSM: Not belonging to sexual minority, SM: Sexual minority
