# Supplementary material for: Moderating effects of self-defined sexual orientation on the relation between social factors and depressive symptoms or suicidal ideation among French young adults
Source: Soc Psychiatry Psychiatr Epidemiol. 2025 Jun 23;60(10):2455–68. doi: 10.1007/s00127-025-02951-y (PMC12449324; doi:10.1007/s00127-025-02951-y)
Supplement: Supplementary file 9 — Supplementary Figure S9. Sensitivity analysis: associations between social factors and depressive symptoms according to sexual orientation (N= 5,544 aged 18–25y; EpiCov study in 2022; weighted and pooled; exclusion of participants who did not wish to report their sexual orientation) [file 127_2025_2951_MOESM9_ESM.pdf]

Factor	n case/N total	.	PR(CI95%)	p value
**Sex at birth**		IR	0.70 (0.49 − 1.00)	0.048
Male:NSM	177/2110		1.00	
Female:NSM	375/2304		1.49 (1.20 − 1.86)	<0.001
Male:SM	75/242		1.00	
Female:SM	169/456		1.03 (0.78 − 1.36)	0.819
**Age category**		IR	0.57 (0.40 − 0.79)	0.001
18 − 21 y:NSM	324/2684		1.00	
22 − 25 y:NSM	283/2117		1.35 (1.06 − 1.71)	0.014
18 − 21 y:SM	163/422		1.00	
22 − 25 y:SM	98/321		0.78 (0.59 − 1.03)	0.084
**Educational attainment**		IR		NS
Higher than bac:NSM	262/2082		1.00	
Bac and lower:NSM	345/2718		1.17 (0.93 − 1.46)	0.183
Higher than bac:SM	96/307		1.00	
Bac and lower:SM	165/436		1.11 (0.83 − 1.48)	0.474
**Employment status**		IR	0.61 (0.39 − 0.96)	0.034
Being employed:NSM	114/1303		1.00	
Not being employed:NSM	493/3498		1.81 (1.36 − 2.40)	<0.001
Being employed:SM	38/141		1.00	
Not being employed:SM	223/602		1.15 (0.81 − 1.64)	0.420
**Perceived financial difficulties**		IR		NS
No:NSM	484/4329		1.00	
Yes:NSM	122/463		1.78 (1.38 − 2.29)	<0.001
No:SM	203/642		1.00	
Yes:SM	56/97		1.54 (1.14 − 2.06)	0.004
**In relationship**		IR		NS
Yes:NSM	193/1488		1.00	
No:NSM	414/3313		1.07 (0.86 − 1.32)	0.546
Yes:SM	75/196		1.00	
No:SM	186/547		0.94 (0.72 − 1.22)	0.629
**Living alone**		IR	0.82 (0.58 − 1.14)	0.235
No:NSM	391/3408		1.00	
Yes:NSM	215/1388		1.32 (1.07 − 1.64)	0.011
No:SM	178/495		1.00	
Yes:SM	82/246		1.06 (0.80 − 1.39)	0.702
**Urban density**		IR		NS
Rural:NSM	134/1200		1.00	
Intermediate:NSM	385/2930		1.00 (0.78 − 1.29)	0.969
Rural:SM	53/156		1.00	
Intermediate:SM	168/482		1.05 (0.77 − 1.44)	0.739
**Urban density**		IR		NS
Rural:NSM	134/1200		1.00	
High−Paris:NSM	88/671		1.13 (0.81 − 1.59)	0.474
Rural:SM	53/156		1.00	
High−Paris:SM	40/105		1.02 (0.66 − 1.58)	0.927
**Discrimination**		IR	0.75 (0.54 − 1.03)	0.076
No:NSM	369/3878		1.00	
Yes:NSM	237/920		2.48 (2.00 − 3.08)	<0.001
No:SM	128/486		1.00	
Yes:SM	133/257		1.88 (1.46 − 2.43)	<0.001

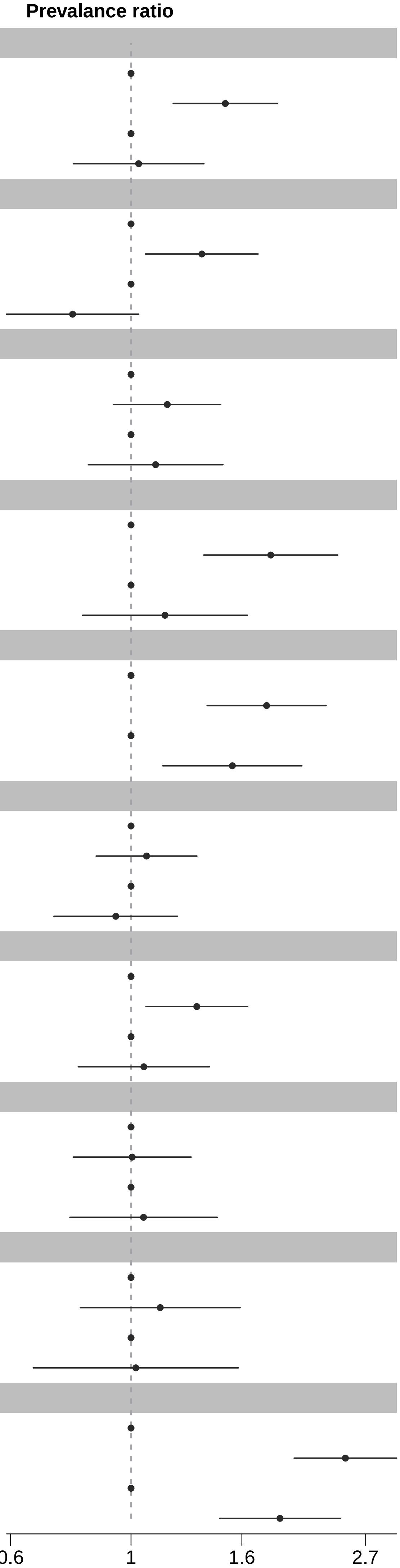

PR: Prevalence ratio, CI: Confidence interaval, NS: Interaction test non significatif in preliminary analysis

NSM: Not belonging to sexual minority, IR: Interaction ratio, SM: Sexual minority
