# Supplementary material for: Moderating effects of self-defined sexual orientation on the relation between social factors and depressive symptoms or suicidal ideation among French young adults
Source: Soc Psychiatry Psychiatr Epidemiol. 2025 Jun 23;60(10):2455–68. doi: 10.1007/s00127-025-02951-y (PMC12449324; doi:10.1007/s00127-025-02951-y)
Supplement: Supplementary file 10 — Supplementary Figure S10. Sensitivity analysis: associations between social factors and suicidal ideation according to sexual orientation (N= 5,544 aged 18–25y; EpiCov study in 2022; weighted and pooled; exclusion of participants who did not wish to report their sexual orientation) [file 127_2025_2951_MOESM10_ESM.pdf]

Factor	n case/N total	.	PR(CI95%)	p value
Sex at birth		IR		NS
Male:NSM	97/2110		1.00	
Female:NSM	161/2304		1.32 (0.96 − 1.81)	0.087
Male:SM	56/242		1.00	
Female:SM	124/456		1.17 (0.82 − 1.67)	0.382
Age category		IR		NS
18 − 21 y:NSM	142/2684		1.00	
22 − 25 y:NSM	134/2117		1.40 (0.96 − 2.05)	0.081
18 − 21 y:SM	108/422		1.00	
22 − 25 y:SM	85/321		1.07 (0.77 − 1.47)	0.698
Educational attainment		IR		NS
Higher than bac:NSM	118/2082		1.00	
Bac and lower:NSM	158/2718		1.29 (0.92 − 1.82)	0.142
Higher than bac:SM	74/307		1.00	
Bac and lower:SM	119/436		1.04 (0.75 − 1.44)	0.811
Employment status		IR	0.60 (0.35 − 1.06)	0.077
Being employed:NSM	55/1303		1.00	
Not being employed:NSM	221/3498		1.97 (1.30 − 2.99)	0.001
Being employed:SM	31/141		1.00	
Not being employed:SM	162/602		1.17 (0.78 − 1.76)	0.445
Perceived fincial difficulties		IR	0.52 (0.30 − 0.91)	0.021
No:NSM	221/4329		1.00	
Yes:NSM	55/463		1.73 (1.17 − 2.56)	0.006
No:SM	161/642		1.00	
Yes:SM	31/97		0.91 (0.61 − 1.36)	0.639
In relationship		IR	0.50 (0.31 − 0.79)	0.003
Yes:NSM	66/1488		1.00	
No:NSM	210/3313		1.63 (1.19 − 2.25)	0.003
Yes:SM	58/196		1.00	
No:SM	135/547		0.82 (0.59 − 1.13)	0.221
Living alone		IR	0.67 (0.42 − 1.07)	0.090
No:NSM	178/3408		1.00	
Yes:NSM	97/1388		1.47 (1.05 − 2.04)	0.023
No:SM	132/495		1.00	
Yes:SM	61/246		0.96 (0.68 − 1.34)	0.797
Urban density		IR		NS
Rural:NSM	51/1200		1.00	
Intermediate:NSM	184/2930		1.27 (0.86 − 1.87)	0.227
Rural:SM	42/156		1.00	
Intermediate:SM	122/482		1.06 (0.72 − 1.56)	0.780
Urban density		IR		NS
Rural:NSM	51/1200		1.00	
High−Paris:NSM	41/671		1.33 (0.81 − 2.18)	0.256
Rural:SM	42/156		1.00	
High−Paris:SM	29/105		0.88 (0.51 − 1.50)	0.634
Discrimination		IR	0.77 (0.49 − 1.19)	0.234
No:NSM	158/3878		1.00	
Yes:NSM	118/920		2.56 (1.88 − 3.50)	<0.001
No:SM	97/486		1.00	
Yes:SM	96/257		1.99 (1.46 − 2.72)	<0.001

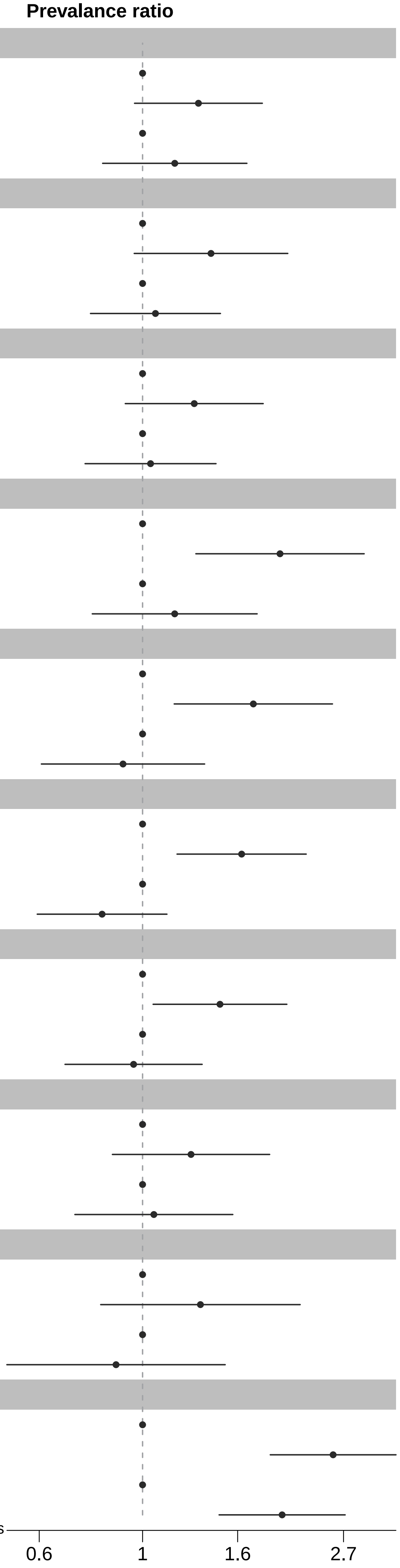

PR: Prevalence ratio, CI: Confidence interaval, NS: Interaction test non significatif in preliminary analysis

NSM: Not belonging to sexual minority, IR: Interaction ratio, SM: Sexual minority
