# Supplementary material for: Moderating effects of self-defined sexual orientation on the relation between social factors and depressive symptoms or suicidal ideation among French young adults
Source: Soc Psychiatry Psychiatr Epidemiol. 2025 Jun 23;60(10):2455–68. doi: 10.1007/s00127-025-02951-y (PMC12449324; doi:10.1007/s00127-025-02951-y)
Supplement: Supplementary file 11 — Supplementary file11 (DOCX 43 kb) [file 127_2025_2951_MOESM11_ESM.docx]

**Supplementary Table S1**. Characteristics of participants aged 18–25y according to detail of sexual orientation (N= 6,337; EpiCov study in 2022; not imputed)

| **Characteristic** | **Heterosexual**, N= 4801^1^ | **95% CI**^2^ | **Homosexual**, N= 120^1^ | **95% CI**^2^ | **Bisexual**, N= 315^1^ | **95% CI**^2^ | **Other**, N= 69^1^ | **95% CI**^2^ | **Undefined**, N= 239^1^ | **95% CI**^2^ | **Does not wish to answer**, N= 793^1^ | **95% CI**^2^ | **p-value**^3^ |
| --- | --- | --- | --- | --- | --- | --- | --- | --- | --- | --- | --- | --- | --- |
| **Depressive symptoms during last 15 days** | |  |  |  |  |  |  |  |  |  |  |  | <0.001 |
| Yes | 13.2% (607) | 11.9%, 14.6% | 27.5% (36) | 18.3%, 39.1% | 40.1% (125) | 33.5%, 47.1% | 37.4% (27) | 23.7%, 53.5% | 28.6% (73) | 22.2%, 36.1% | 10.7% (90) | 8.21%, 13.9% |  |
| **Suicidal ideation during last 12 months** | |  |  |  |  |  |  |  |  |  |  |  | <0.001 |
| Yes | 5.6% (276) | 4.83%, 6.51% | 18.4% (32) | 12.1%, 27.1% | 29.4% (95) | 23.7%, 35.9% | 19.8% (15) | 10.5%, 34.0% | 18.6% (51) | 13.4%, 25.2% | 4.7% (42) | 3.32%, 6.67% |  |
| **Sex** |  |  |  |  |  |  |  |  |  |  |  |  | <0.001 |
| Female | 43.2% (2,304) | 41.3%, 45.1% | 29.5% (42) | 19.9%, 41.4% | 69.4% (227) | 62.3%, 75.8% | 43.8% (34) | 28.0%, 61.0% | 61.6% (153) | 52.4%, 70.1% | 49.5% (389) | 44.1%, 54.9% |  |
| **Age category** |  |  |  |  |  |  |  |  |  |  |  |  | 0.009 |
| 18–21 y | 47.2% (2,117) | 45.3%, 49.2% | 54.9% (62) | 41.9%, 67.2% | 35.7% (112) | 29.5%, 42.5% | 46.2% (28) | 30.2%, 63.0% | 51.6% (119) | 43.2%, 59.9% | 41.0% (319) | 36.1%, 46.0% |  |
| 22–25 y | 52.8% (2,684) | 50.8%, 54.7% | 45.1% (58) | 32.8%, 58.1% | 64.3% (203) | 57.5%, 70.5% | 53.8% (41) | 37.0%, 69.8% | 48.4% (120) | 40.1%, 56.8% | 59.0% (474) | 54.0%, 63.9% |  |
| **Educational attainment higher than high school** | |  |  |  |  |  |  |  |  |  |  |  | <0.001 |
| Yes | 39.8% (2,082) | 38.0%, 41.6% | 54.0% (68) | 40.4%, 67.1% | 34.0% (117) | 27.9%, 40.6% | 26.8% (19) | 15.7%, 41.7% | 38.1% (103) | 30.8%, 46.0% | 30.5% (274) | 26.4%, 34.9% |  |
| **Being employed** |  |  |  |  |  |  |  |  |  |  |  |  | 0.012 |
| Yes | 30.2% (1,303) | 28.4%, 32.0% | 28.4% (26) | 17.7%, 42.2% | 17.2% (50) | 12.7%, 22.9% | 25.4% (21) | 15.0%, 39.6% | 21.4% (44) | 15.4%, 28.9% | 28.9% (211) | 24.4%, 33.8% |  |
| **Perceived financial difficulties** |  |  |  |  |  |  |  |  |  |  |  |  | 0.089 |
| Yes | 11.2% (463) | 9.93%, 12.6% | 13.9% (17) | 7.79%, 23.4% | 12.0% (41) | 8.49%, 16.8% | 20.2% (12) | 10.1%, 36.2% | 15.5% (27) | 9.44%, 24.4% | 15.0% (121) | 12.1%, 18.3% |  |
| **In a relationship** |  |  |  |  |  |  |  |  |  |  |  |  | <0.001 |
| Yes | 28.3% (1,488) | 26.7%, 30.0% | 19.6% (30) | 12.8%, 28.8% | 31.7% (104) | 25.5%, 38.5% | 23.4% (17) | 12.9%, 38.5% | 18.6% (45) | 13.5%, 25.0% | 10.1% (88) | 7.66%, 13.2% |  |
| **Living alone** |  |  |  |  |  |  |  |  |  |  |  |  | <0.001 |
| Yes | 27.6% (1,388) | 26.0%, 29.3% | 32.4% (43) | 21.9%, 45.1% | 34.6% (110) | 28.3%, 41.4% | 29.4% (18) | 17.4%, 45.1% | 32.5% (75) | 24.7%, 41.4% | 18.3% (170) | 15.2%, 22.0% |  |
| **Urban density** |  |  |  |  |  |  |  |  |  |  |  |  | 0.962 |
| Rural | 23.6% (1,200) | 22.0%, 25.2% | 20.8% (21) | 9.90%, 38.7% | 22.7% (64) | 16.9%, 29.6% | 28.2% (20) | 16.8%, 43.3% | 20.9% (51) | 15.4%, 27.7% | 23.3% (196) | 19.4%, 27.7% |  |
| Intermediate | 60.5% (2,930) | 58.6%, 62.3% | 59.4% (79) | 44.6%, 72.6% | 62.3% (212) | 54.9%, 69.2% | 51.1% (41) | 34.8%, 67.1% | 59.5% (150) | 50.8%, 67.6% | 59.3% (482) | 54.2%, 64.1% |  |
| High - Paris area | 15.9% (671) | 14.5%, 17.5% | 19.8% (20) | 10.7%, 33.7% | 15.0% (39) | 10.1%, 21.6% | 20.7% (8) | 7.78%, 44.7% | 19.6% (38) | 12.7%, 29.1% | 17.4% (115) | 13.6%, 22.1% |  |
| **Experience of discrimination in the last 5 y** | |  |  |  |  |  |  |  |  |  |  |  | <0.001 |
| Yes | 19.2% (920) | 17.7%, 20.7% | 35.8% (53) | 25.3%, 47.8% | 33.3% (109) | 27.0%, 40.3% | 29.3% (23) | 17.9%, 44.0% | 31.3% (72) | 24.2%, 39.3% | 21.2% (169) | 17.2%, 25.7% |  |
| **Chronic somatic or mental conditions** | |  |  |  |  |  |  |  |  |  |  |  | 0.004 |
| Yes | 23.9% (1,113) | 22.3%, 25.6% | 34.7% (41) | 22.3%, 49.6% | 30.5% (105) | 24.8%, 36.8% | 44.7% (27) | 28.6%, 61.9% | 30.5% (74) | 23.7%, 38.2% | 22.0% (179) | 18.3%, 26.2% |  |
| **History of mental disorders diagnosis** | |  |  |  |  |  |  |  |  |  |  |  | <0.001 |
| Yes | 5.8% (304) | 5.06%, 6.74% | 17.1% (30) | 11.1%, 25.4% | 23.1% (84) | 18.2%, 29.0% | 19.8% (17) | 10.8%, 33.6% | 18.6% (37) | 11.8%, 28.2% | 6.6% (61) | 4.78%, 9.03% |  |
| **Web questionnaire type** |  |  |  |  |  |  |  |  |  |  |  |  | 0.020 |
| Yes | 69.2% (3,428) | 67.4%, 70.9% | 74.6% (96) | 60.8%, 84.8% | 74.9% (244) | 68.1%, 80.6% | 82.0% (54) | 68.8%, 90.4% | 76.7% (187) | 68.4%, 83.3% | 75.3% (583) | 71.1%, 79.0% |  |
| ^1^% (n (unweighted)) |  |  |  |  |  |  |  |  |  |  |  |  |  |
| ^2^CI = Confidence Interval |  |  |  |  |  |  |  |  |  |  |  |  |  |
| ^3^chi-squared test with Rao & Scott's second-order correction | | |  |  |  |  |  |  |  |  |  |  |  |

Sex, education attainment, being employed, perceived financial difficulties, living alone, experience of discrimination, chronic somatic and mental conditions, history of mental disorders diagnosis contained 545, 2, 1, 24, 8, 9, 76, and 2 missing values, respectively.

NSM: Not belonging to the Sexual Minority, SM: Sexual Minority

**Supplementary Table S2**. Details of variables related to social factors

| Variable | Source | Question in questionnaires | Modality detail |
| --- | --- | --- | --- |
| Sex | INSEE | - | Male Female |
| Age category | INSEE | - | 18–21 y 22–25 y |
| Education attainment | EpiCov | What degree(s) do you have? | List of 13 possibilities ≤ French baccalaureate > French baccalaureate |
| Employment status | EpiCov | What is your current main situation? | Being employed: Employment (salaried or self-employed, including helping someone to work, short-time working) Not being employed: apprenticeship or paid internship/studies (students) or unpaid internship/unemployment/housewife or house husband/others |
| Perceived financial difficulties | EpiCov | Financially in your household would you rather say that today… | Yes: You can hardly do it/you can't do it without going into debt (or using consumer credit) No: You are comfortable/it's OK/it's right/it's tight/you have to be careful |
| In relationship | EpiCov | Are you currently in a relationship? | Yes No |
| Living alone | EpiCov, DREES | Number of people living in household calculated by DREES | Yes: 1 No: ≥2 |
| Urban density | INSEE | - | Rural: <2,000 urban units Intermediate: 2,000–1,999,999 urban units High: 2,000,000 urban units or Paris area |
| Experience of discrimination | EpiCov | In the last 5 years, do you think you have experienced unequal treatment or discrimination? This may have occurred at work, at home, with a healthcare professional, in hospital, at school, in the administration, on the street, etc. | Yes: Often/sometimes No: Never |
| Chronic somatic or mental conditions | EpiCov | Have you been limited, for at least 6 months, because of a health problem, in the activities people usually do? | Yes: Strongly limited/limited but not strongly No |
|  | EpiCov | Do you have an illness or health problem that is chronic or long-lasting? | Yes  No |
| History of mental disorders diagnosis | EpiCov | During your lifetime (at summer 2021) and since summer 2021 (et the 4th wave), has a doctor ever told you that you have a psychiatric or psychological disorder or an addiction? | Yes  No |
| Questionnaire type | EpiCov | - | Web: Computer-assisted web interviews Telephone: Computer-assisted telephone interviews |
| DREES: French Ministry for Research, Direction de la Recherche, des Etudes, de l’Evaluation et des Statistiques; INSEE: National Institute for Statistics and Economic Studies | | | |

**Supplementary Table S3**. Comparison of characteristics of included and excluded participants aged 18–25 y (EpiCov study in 2022, not imputed)

| **Characteristic** | **Excluded**,  N= 1430^1^ | **95% CI**^2^ | **Included**,  N= 6337^1^ | **95% CI**^2^ | **p-value**^3^ |
| --- | --- | --- | --- | --- | --- |
| **Depressive symptoms during last 15 days** |  |  |  |  | <0.001 |
| Yes | 21.4% (317) | 18.7%, 24.4% | 15.1% (958) | 14.0%, 16.3% |  |
| **Suicidal ideation during last 12 months** |  |  |  |  | 0.018 |
| Yes | 5.1% (68) | 3.85%, 6.83% | 7.4% (511) | 6.65%, 8.23% |  |
| **Sex** |  |  |  |  | <0.001 |
| Female | 54.3% (843) | 50.6%, 58.1% | 45.6% (3,149) | 43.9%, 47.3% |  |
| **Age category** |  |  |  |  | <0.001 |
| 18–21 y | 58.6% (844) | 55.0%, 62.0% | 46.2% (2,757) | 44.5%, 47.9% |  |
| 22–25 y | 41.4% (586) | 38.0%, 45.0% | 53.8% (3,580) | 52.1%, 55.5% |  |
| **Educational attainment higher than high school** | |  |  |  | 0.162 |
| Yes | 41.0% (643) | 37.6%, 44.5% | 38.4% (2,663) | 36.8%, 39.9% |  |
| **Being employed** |  |  |  |  | <0.001 |
| Yes | 44.8% (632) | 41.3%, 48.4% | 29.0% (1,655) | 27.5%, 30.6% |  |
| **Perceived financial difficulties** |  |  |  |  | 0.002 |
| Yes | 16.4% (220) | 13.9%, 19.4% | 12.1% (681) | 10.9%, 13.3% |  |
| **In a relationship** |  |  |  |  | 0.005 |
| Yes | 18.8% (115) | 15.1%, 23.2% | 25.4% (1,772) | 24.1%, 26.8% |  |
| **Living alone** |  |  |  |  | <0.001 |
| Yes | 7.9% (127) | 6.31%, 9.76% | 26.9% (1,804) | 25.5%, 28.4% |  |
| **Urban density** |  |  |  |  | <0.001 |
| Rural | 27.8% (416) | 24.7%, 31.0% | 23.4% (1,552) | 22.0%, 24.8% |  |
| Intermediate | 61.4% (891) | 57.9%, 64.9% | 60.2% (3,894) | 58.6%, 61.9% |  |
| High - Paris area | 10.8% (123) | 8.65%, 13.4% | 16.4% (891) | 15.1%, 17.8% |  |
| **Experience of discrimination in the last 5 y** |  |  |  |  | 0.027 |
| Yes | 24.5% (368) | 21.7%, 27.6% | 20.9% (1,346) | 19.6%, 22.3% |  |
| **Chronic health conditions** |  |  |  |  | 0.202 |
| Yes | 26.9% (377) | 23.8%, 30.3% | 24.6% (1,539) | 23.2%, 26.1% |  |
| **History of mental disorders diagnosis** |  |  |  |  | 0.008 |
| Yes | 10.4% (167) | 8.42%, 12.8% | 7.5% (533) | 6.76%, 8.40% |  |
| **Web questionnaire type** |  |  |  |  | 0.117 |
| Yes | 73.8% (1,102) | 70.3%, 77.0% | 70.8% (4,592) | 69.2%, 72.3% |  |
| ^1^% (n (unweighted)) | | | | | |
| ^2^CI = Confidence Interval | | | | | |
| ^3^chi-squared test with Rao & Scott's second-order correction  Depressive symptoms, suicidal ideation, sex, education attainment, being employed, perceived financial difficulties, in relationship, living alone, experience of discrimination, chronic health conditions, history of mental disorders diagnosis contained 17, 337, 640, 3, 1 32, 912, 10, 14, 93, and 11 missing values, respectively. | | | | | |

**Supplementary Table S4**. Comparison of characteristics of participants aged 18–25 y according to depressive symptoms (EpiCov study in 2022, not imputed)

| **Characteristic** | **Depressive symptoms: No** | **95% CI**^2^ | **Depressive symptoms: Yes** | **95% CI**^2^ | **p-value**^3^ |
| --- | --- | --- | --- | --- | --- |
|  | N = 5,379^1^ |  | N = 958^1^ |  |  |
| **Sexual orientation** |  |  |  |  | <0.001 |
| Sexual minority | 8.60% (482) | 7.61%, 9.70% | 24.7% (261) | 21.5%, 28.2% |  |
| **Suicidal ideation during last 12 months** | |  |  |  | <0.001 |
| Yes | 2.71% (168) | 2.25%, 3.25% | 33.8% (343) | 30.0%, 37.8% |  |
| **Sex** |  |  |  |  | <0.001 |
| Female | 43.2% (2,546) | 41.3%, 45.0% | 59.4% (603) | 54.7%, 64.0% |  |
| **Age category** |  |  |  |  | 0.825 |
| 18 - 21y | 46.1% (2,340) | 44.3%, 47.9% | 46.6% (417) | 42.4%, 50.9% |  |
| 22 - 25y | 53.9% (3,039) | 52.1%, 55.7% | 53.4% (541) | 49.1%, 57.6% |  |
| **Educational attainment higher than high school** | |  |  |  | 0.342 |
| Yes | 38.7% (2,270) | 37.0%, 40.4% | 36.6% (393) | 32.8%, 40.6% |  |
| **Being employed** |  |  |  |  | <0.001 |
| Yes | 30.7% (1,482) | 28.9%, 32.4% | 19.9% (173) | 16.6%, 23.8% |  |
| **Perceived financial difficulties** |  |  |  |  | <0.001 |
| Yes | 10.3% (482) | 9.17%, 11.6% | 21.9% (199) | 18.5%, 25.6% |  |
| **In a relationship** |  |  |  |  | 0.404 |
| Yes | 25.2% (1,489) | 23.7%, 26.7% | 26.8% (283) | 23.4%, 30.6% |  |
| **Living alone** |  |  |  |  | 0.002 |
| Yes | 26.0% (1,478) | 24.5%, 27.5% | 32.3% (326) | 28.5%, 36.3% |  |
| **Urban density** |  |  |  |  | 0.679 |
| Rural | 23.7% (1,340) | 22.2%, 25.2% | 21.9% (212) | 18.5%, 25.7% |  |
| Intermediate | 60.1% (3,288) | 58.2%, 61.9% | 61.1% (606) | 56.8%, 65.3% |  |
| High - Paris area | 16.3% (751) | 14.9%, 17.8% | 17.0% (140) | 13.8%, 20.7% |  |
| **Experience of discrimination in the last 5 y** | |  |  |  | <0.001 |
| Yes | 17.0% (934) | 15.7%, 18.4% | 42.8% (412) | 38.7%, 47.1% |  |
| **Chronic health conditions** |  |  |  |  | <0.001 |
| Yes | 21.6% (1,155) | 20.1%, 23.1% | 42.0% (384) | 37.8%, 46.3% |  |
| **History of mental disorders diagnosis** | |  |  |  | <0.001 |
| Yes | 4.88% (286) | 4.17%, 5.70% | 22.5% (247) | 19.4%, 25.8% |  |
| **Web questionnaire type** |  |  |  |  | 0.005 |
| Yes | 69.8% (3,842) | 68.2%, 71.5% | 76.1% (750) | 72.1%, 79.7% |  |
| ^1^% (n (unweighted)) | | | | | |
| ^2^CI = Confidence Interval | | | | | |
| ^3^Pearson's X^2: Rao & Scott adjustment | | | | | |

Sex, education attainment, being employed, perceived financial difficulties, living alone, experience of discrimination, chronic somatic and mental conditions, history of mental disorders diagnosis contained 545, 2, 1, 24, 8, 9, 76, and 2 missing values, respectively.

**Supplementary Table S5**. Comparison of characteristics of participants aged 18–25 y according to suicidal ideation (EpiCov study in 2022, not imputed)

| **Characteristic** | **Suicidal ideation: No** | **95% CI**^2^ | **Suicidal ideation: Yes** | **95% CI**^2^ | **p-value**^3^ |
| --- | --- | --- | --- | --- | --- |
|  | N = 5,826^1^ |  | N = 511^1^ |  |  |
| **Sexual orientation** |  |  |  |  | <0.001 |
| Sexual minority | 9.18% (550) | 8.21%, 10.3% | 34.2% (193) | 29.5%, 39.3% |  |
| **Depressive symptoms during last 15 days** | |  |  |  | <0.001 |
| Yes | 10.8% (615) | 9.74%, 12.0% | 68.9% (343) | 63.8%, 73.6% |  |
| **Sex** |  |  |  |  | <0.001 |
| Female | 44.5% (2,835) | 42.7%, 46.3% | 58.6% (314) | 52.8%, 64.2% |  |
| **Age category** |  |  |  |  | 0.756 |
| 18 - 21y | 46.1% (2,523) | 44.4%, 47.9% | 47.0% (234) | 41.6%, 52.5% |  |
| 22 - 25y | 53.9% (3,303) | 52.1%, 55.6% | 53.0% (277) | 47.5%, 58.4% |  |
| **Educational attainment higher than high school** | | |  |  | 0.166 |
| Yes | 38.6% (2,457) | 37.0%, 40.3% | 34.9% (206) | 30.2%, 40.0% |  |
| **Being employed** |  |  |  |  | <0.001 |
| Yes | 29.9% (1,562) | 28.3%, 31.6% | 18.2% (93) | 14.5%, 22.7% |  |
| **Perceived financial difficulties** | |  |  |  | <0.001 |
| Yes | 11.5% (589) | 10.3%, 12.8% | 19.0% (92) | 15.0%, 23.7% |  |
| **In a relationship** |  |  |  |  | 0.261 |
| Yes | 25.6% (1,643) | 24.2%, 27.1% | 23.0% (129) | 19.0%, 27.5% |  |
| **Living alone** |  |  |  |  | 0.020 |
| Yes | 26.5% (1,632) | 25.0%, 28.0% | 32.6% (172) | 27.6%, 38.0% |  |
| **Urban density** |  |  |  |  | 0.247 |
| Rural | 23.7% (1,449) | 22.3%, 25.2% | 19.6% (103) | 15.6%, 24.2% |  |
| Intermediate | 60.0% (3,567) | 58.3%, 61.7% | 62.8% (327) | 57.4%, 67.9% |  |
| High - Paris area | 16.3% (810) | 14.9%, 17.7% | 17.6% (81) | 13.8%, 22.2% |  |
| **Experience of discrimination in the last 5 y** | |  |  |  | <0.001 |
| Yes | 19.0% (1,113) | 17.7%, 20.4% | 45.3% (233) | 40.0%, 50.8% |  |
| **Chronic health conditions** |  |  |  |  | <0.001 |
| Yes | 23.3% (1,335) | 21.9%, 24.9% | 41.2% (204) | 35.8%, 46.8% |  |
| **History of mental disorders diagnosis** | |  |  |  | <0.001 |
| Yes | 5.37% (337) | 4.66%, 6.19% | 34.6% (196) | 29.8%, 39.8% |  |
| **Web questionnaire type** |  |  |  |  | 0.003 |
| Yes | 70.2% (4,181) | 68.6%, 71.8% | 78.3% (411) | 73.3%, 82.6% |  |
| ^1^% (n (unweighted)) | | | | | |
| ^2^CI = Confidence Interval | | | | | |
| ^3^Pearson's X^2: Rao & Scott adjustment | | | | | |

Sex, education attainment, being employed, perceived financial difficulties, living alone, experience of discrimination, chronic somatic and mental conditions, history of mental disorders diagnosis contained 545, 2, 1, 24, 8, 9, 76, and 2 missing values, respectively.
